# Contrasting glacier mass balance changes in High Mountain Asia from 1975 to 2013

**DOI:** 10.1093/nsr/nwag486

**Published:** 2026-08-12

**Authors:** Yushan Zhou, Yingzheng Wang, Donghai Zheng, Wenfeng Chen, Guoqing Zhang, Bo Su, Bin Cao, Jun Hu, Zhiwei Li, Xin Li

**Affiliations:** School of Geosciences and Info-Physics, Central South University, China; National Tibetan Plateau Data Center, State Key Laboratory of Tibetan Plateau Earth System, Environment and Resources (TPESER), Institute of Tibetan Plateau Research, Chinese Academy of Sciences, China; National Tibetan Plateau Data Center, State Key Laboratory of Tibetan Plateau Earth System, Environment and Resources (TPESER), Institute of Tibetan Plateau Research, Chinese Academy of Sciences, China; National Tibetan Plateau Data Center, State Key Laboratory of Tibetan Plateau Earth System, Environment and Resources (TPESER), Institute of Tibetan Plateau Research, Chinese Academy of Sciences, China; National Tibetan Plateau Data Center, State Key Laboratory of Tibetan Plateau Earth System, Environment and Resources (TPESER), Institute of Tibetan Plateau Research, Chinese Academy of Sciences, China; Stockholm Resilience Centre, Stockholm University, Sweden; Regional Climate Group, Department of Earth Sciences, University of Gothenburg, Sweden; National Tibetan Plateau Data Center, State Key Laboratory of Tibetan Plateau Earth System, Environment and Resources (TPESER), Institute of Tibetan Plateau Research, Chinese Academy of Sciences, China; School of Geosciences and Info-Physics, Central South University, China; School of Geosciences and Info-Physics, Central South University, China; National Tibetan Plateau Data Center, State Key Laboratory of Tibetan Plateau Earth System, Environment and Resources (TPESER), Institute of Tibetan Plateau Research, Chinese Academy of Sciences, China

**Keywords:** glacier mass balance, High Mountain Asia, geodetic method

High Mountain Asia (HMA) contains the largest glacier storage outside the polar regions, and is one of the most critical ‘water tower’ in the world [1]. The snow and glacier meltwater from this region directly impacts the livelihoods of 250 million people downstream, as well as industrial and agricultural development, ecological systems, hydropower generation, and other socio-economic activities [2]. In the context of a warming climate, increasing glacier instability presents multifaceted challenges and threats to the region, especially with the rise in human activities.

In response, significant efforts have been made to monitor glacier mass changes across the region, particularly through the use of satellite data [3,4]. Affected by the availability of satellite data, numerous studies have focused on short-term (e.g. decadal or shorter) changes over the past two decades (since 2000) [3,5]. However, with the release and utilization of archived historical data (e.g. KH-4/9 satellite images), long-term (multi-decadal) monitoring of glacier mass change has become more feasible, with studies extending as far back as the 1970s and even the 1960s [6]. Despite these advancements, most studies have concentrated on regional or mountain-scale assessments, and differences in data processing methods (including uncertainty assessment) have led to challenges in making reliable comparisons across studies [7]. Consequently, the long-term evolution of glaciers across the whole HMA remains poorly understood.

To address this gap, we first processed all available KH-9 stereo image pairs acquired between 1973 and 1980 over the glacierized areas of HMA to generate 145 KH-9 digital elevation models (DEMs). These DEMs were then combined with the SRTM (in 2000) and the Copernicus DEM (for 2011–2015) using a harmonized data processing framework. This enabled, for the first time, the reconstruction of glacier mass balance across the entire HMA over the multi-decadal period from 1975 to 2013 using the geodetic method. Valid observations covered 59% and 91% of the total glacierized area in HMA during the two study periods, respectively, and glaciers smaller than 0.5 km^2^ were excluded from the analysis. We further investigated the climatic drivers of the contrasting glacier mass changes across HMA. Details of the data processing methods and uncertainty assessment are provided in the Supplementary Methods.

From the mid-1970s to 2000, HMA glaciers experienced an overall mass change rate of −0.13 ± 0.23 m w.e./a (equivalent to −12.80 ± 22.64 Gt/a), with pronounced spatial heterogeneity (Fig. 1a, Tables S1 and S2). The most negative glacier mass change rates were observed in the Gangdise (−0.42 ± 0.16 m w.e./a) and western Tianshan (−0.38 ± 0.24 m w.e./a), followed by the south-eastern margin of HMA (i.e. the Hengduan and central-eastern Himalaya ranges), the central-eastern Tianshan, and the Pamir–Alay regions, where glacier mass change rates ranged from −0.27 ± 0.21 to −0.21 ± 0.34 m w.e./a. In contrast, the western Kunlun and surrounding areas exhibited the smallest glacier mass changes, with an approximately balanced mass budget in the western Kunlun (0.00 ± 0.24 m w.e./a) and near-balanced conditions in the Karakoram–Pamir region (from −0.06 ± 0.22 to −0.02 ± 0.34 m w.e./a). These results are consistent with previous findings of the western Kunlun–Karakorum–Pamir anomaly centered on the western Kunlun mountain [8], but are supported here by a larger number of observations. Glaciers in the western Himalaya, Hindu Kush, and eastern Kunlun mountains experienced relatively small mass change rates, ranging from −0.12 ± 0.31 to −0.09 ± 0.27 m w.e./a. Other mountain regions experienced moderate glacier mass change rates, ranging from −0.19 ± 0.20 to −0.15 ± 0.14 m w.e./a. More details are provided in supplementary materials (Fig. 1a, Table S1, and Figs S1 and S2).

**Figure 1.**
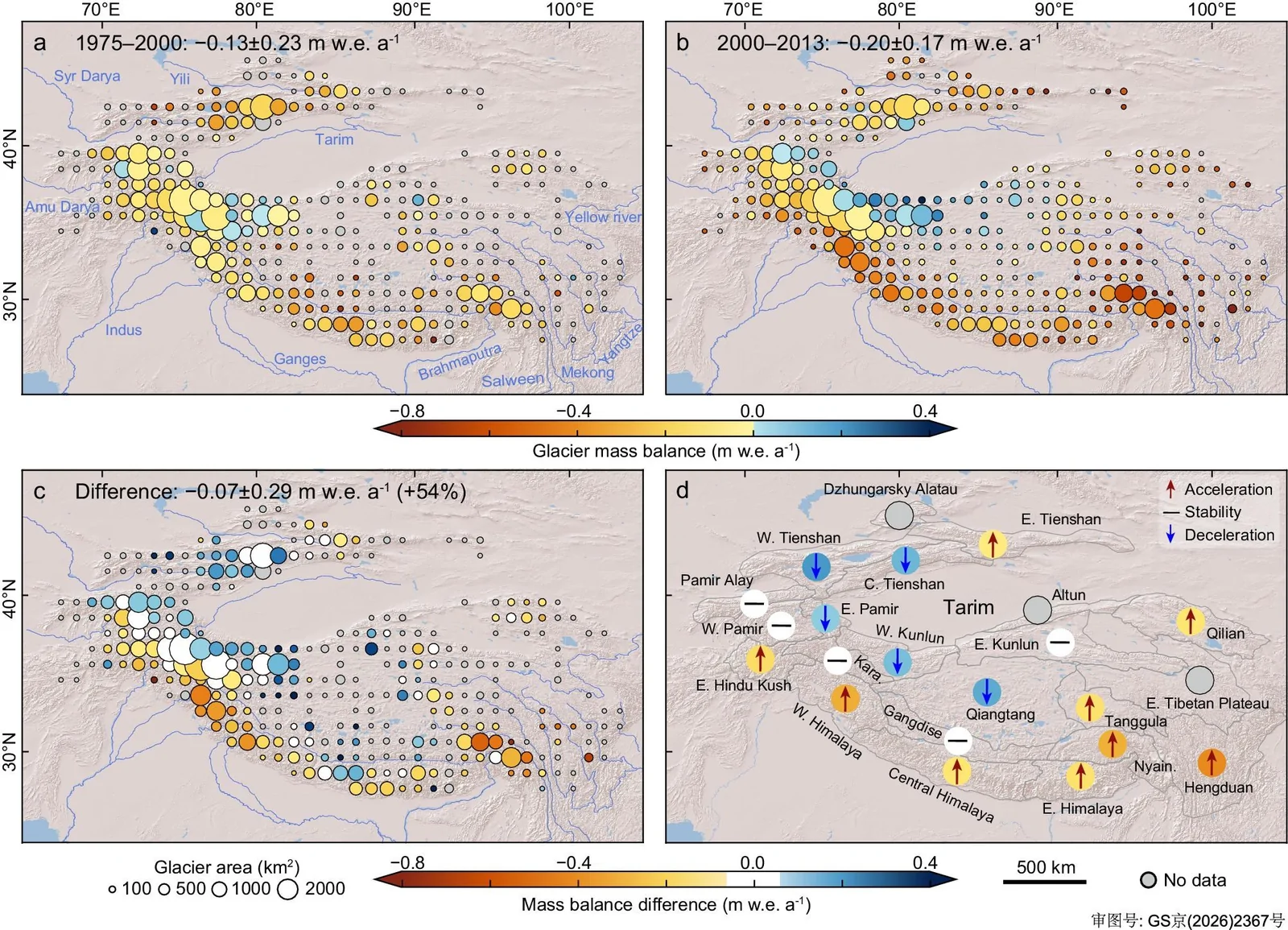
Changes in glacier mass balance over four decades in HMA. Glacier mass balance at the 1° geographic grid for the periods 1975–2000 (a) and for 2000–2013 (b), respectively. Changes in glacier mass balance at the geographic grid (c) and mountain scales (d), respectively.

Between 2000 and 2013, the HMA glacier mass change rate was −0.20 ± 0.17 m w.e./a (a total mass change of −19.69 ± 16.73 Gt/a), with greater spatial variability than during the previous study period (Fig. 1b, and Tables S1 and S2). The most negative glacier mass change rates occurred in the Hengduan mountain (−0.65 ± 0.21 m w.e./a), followed by the Nyainqentanglha mountain (−0.49 ± 0.26 m w.e./a) (Fig. 1b). Pronounced glacier mass loss was also observed along the northern (the eastern Tianshan and Dzhungarsky Alatau range), eastern (eastern Tibetan mountains), and southern (the Gangdise–Himalaya ranges) margins of the HMA, with glacier mass change rates ranging from −0.42 ± 0.30 to −0.33 ± 0.15 m w.e./a. In addition, glaciers in the western Tianshan, Pamir Alay, Hindu Kush, Qilian, and Tanggula mountains experienced moderate mass loss, with glacier mass change rates ranging from −0.28 ± 0.22 to −0.16 ± 0.21 m w.e./a. Relatively small glacier mass loss occurred in the central Tianshan, Altun, western Pamir, and Karakoram mountains, where glacier mass change rates ranged from −0.13 ± 0.12 to −0.09 ± 0.22 m w.e./a. In contrast, glaciers in the Tibetan Interior (i.e. Qiangtang) and eastern Kunlun mountains experienced slightly negative mass balance or approximately balanced state, with glacier mass change rates of −0.03 ± 0.10 and −0.05 ± 0.11 w.e./a, respectively. Notably, glaciers along the southern margin of Tarim Basin (the eastern Pamir and western Kunlun Mountains) experienced slight mass gain, with glacier mass change rates of 0.06 ± 0.13 and 0.10 ± 0.17 w.e./a, respectively. These results suggest that the so-called Karakoram anomaly, previously attributed to glaciers in the western Kunlun–Karakorum–Pamir ranges, may instead be centered around the circum-Tarim Basin, as more clearly illustrated by the glacier-level mass balance map (Fig. S3).

Comparisons between the two sub-periods reveal rapid and complex spatio-temporal variability in glacier mass change across HMA (Fig. 1c, d and Table S1). A significance threshold of ±0.06 m w.e./a was adopted to identify significant changes in glacier mass balance. Overall, glacier mass loss in HMA intensified, with the mass balance changing from −0.13 ± 0.23 to −0.20 ± 0.17 m w.e./a, representing a 54% increase in the rate of mass loss after 2000. In the following, changes in glacier mass balance refer to the difference between the two periods (2000–2013 minus 1975–2000), where negative values indicate an acceleration in mass loss and positive values indicate a deceleration. Specifically, the strongest acceleration in glacier mass loss occurred in Hengduan Mountains, with a change in mass balance of −0.42 m w.e./a, followed by the western Himalayas (−0.33 m w.e./a) and the Nyainqentanglha Mountains (−0.31 m w.e./a). A moderate acceleration was observed in the Hindu Kush (−0.16 m w.e./a), while slight accelerations ranging from −0.12 to −0.08 m w.e./a. occurred in the eastern Tianshan, Qilian, Tanggula, and central-eastern Himalaya. In contrast, glaciers in the central Tianshan, the Tibetan Interior, and the western Tianshan experienced a deceleration in mass loss, with mass balance changes of 0.14, 0.16, and 0.20 m w.e./a, respectively. No significant changes were observed in the Pamir Alay, western Pamir, Karakoram, eastern Kunlun, and Gangdise mountains, where mass balance changes remained within ±0.06 m w.e./a, indicating a steady or unchanged rate of mass change. It is worth noting that these mountain-scale steady states represent the average condition of glaciers within each region; however, spatial variations in glacier change still exist within these areas (i.e. some glaciers are accelerating, decelerating, or remaining stable), such as in the Karakoram, Gangdise, and Eastern Kunlun ranges (Fig. 1c). Notably, in the eastern Pamir and western Kunlun ranges, glaciers shifted from near equilibrium to net mass gain, with regional mass balances changing from −0.02 ± 0.34 and 0.00 ± 0.24 during 1975–2000 to 0.06 ± 0.13 and 0.10 ± 0.17 m w.e./a during 2000–2013, respectively (Table S1).

Furthermore, we compared our regional estimates with those reported in previous studies (Fig. S4, Table S3). For the period 1975–2000, our estimates are generally consistent with existing local-scale findings, but this study covers a much larger glacierized areas for each sub-region. For the period 2000–2013, our results are overall in agreement with those of Hugonnet *et al.* (Ref. [9]), that is, −0.20 ± 0.17 m we./a vs. −0.22 ± 0.05 m w.e./a, and the majority of sub-regions show comparable results with existing estimates, with the exception of the Pamir Alay, Hindu Kush, and western Himalaya mountains (Table S1).

The multi-decadal changes in air temperature and precipitation based on ERA5 reanalysis dataset exhibit trends that are consistent with the observed glacier changes (Fig. S5). All mountainous regions within HMA experienced varying degrees of warming (Figs S6 and S7). Partition clustering analysis reveals an almost consistent trend in mean annual air temperature (MAAT) anomaly across the westerly and monsoon zones, as well as all sub-regions (Fig. S5c). In contrast, mean annual precipitation (MAP) variations show significant spatial divergence, and changes in cumulative standardized precipitation (CSP) around 2000 align well with variations in glacier mass balance in the westerly and monsoon zones, respectively. A rising CSP corresponds to a more positive mass balance, while a falling CSP corresponds to a more negative mass balance (Fig. S5d and e).

Additionally, combining changes in MAP and solid precipitation (SP) reveals a complex spatial pattern of precipitation phase transitions (i.e. between solid and liquid precipitation) (Fig. S6b). To explore the response of glacier mass change to different climate zones, we conducted a stepwise analysis of variance for changes in climatic variables and glacier mass change. The results show that changes in liquid precipitation (LP), SP, and summer air temperature account for 54%, 11%, and 8% of the glacier mass change, respectively, in regions affected by westerlies (Fig. S5f). In contrast, in regions influenced by the summer monsoon, MAAT, SP, summer air temperature, and LP contributed to 39%, 36%, 12%, and 4%, respectively, to the glacier mass change (Fig. S5f). This is generally consistent with field-based observations in the southeastern Tibet Plateau [10]. Based on these findings, we conclude that multi-decadal variations in glacier mass balance within the HMA can largely be explained by regional climate change.

In summary, this study presents a spatiotemporally resolved reconstruction of glacier mass balance across HMA from 1975 to 2013, providing a new observational benchmark for the calibration and validation of physics-based glacier and hydrological simulations. Additionally, it offers a comprehensive overview of the long-term evolution of glacier mass balance across the entire HMA, revealing rapid and complex changes occurring over just a few decades. The findings underscore the urgent need for more extensive and long-term monitoring with high spatio-temporal resolution to track the ongoing transformation of glaciers and its potential impacts on water resources, ecosystems, and socio-economic activities. Such monitoring will enable the timely detection of regions undergoing anomalous changes particularly those prone to significant melting, and will offer valuable insights to support governments in formulating effective climate adaptation and mitigation strategies.

## Supplementary Material

nwag486_Supplemental_File
