# Supplementary material for: Contrasting glacier mass balance changes in High Mountain Asia from 1975 to 2013
Source: Natl Sci Rev. 2026 Aug 12;13(18):nwag486. doi: 10.1093/nsr/nwag486 (PMC13602414; doi:10.1093/nsr/nwag486)
Supplement: Supplementary file 1 [file nwag486_supplemental_file.pdf]

Supplementary information for

Contrasting glacier mass balance changes in High Mountain Asia from 1975 to 2013

Yushan Zhou[1†], Yingzheng Wang[2†], Donghai Zheng*[2], Wenfeng Chen[2], Guoqing Zhang[2], Bo Su[3,4], Bin Cao[2], Jun Hu[1], Zhiwei Li[1], Xin Li*[2]

1. School of Geosciences and Info-Physics, Central South University, Changsha, China
2. National Tibetan Plateau Data Center, State Key Laboratory of Tibetan Plateau Earth System, Environment and Resources (TPESER), Institute of Tibetan Plateau Research, Chinese Academy of Sciences, Beijing, China
3. Stockholm Resilience Centre, Stockholm University, Stockholm, Sweden
4. Regional Climate Group, Department of Earth Sciences, University of Gothenburg, Gothenburg, Sweden

†: These authors contributed equally to this paper.

***Corresponding author:** xinli@itpcas.ac.cn, zhengd@itpcas.ac.cn

The file includes:

Supplementary Methods

Supplementary Figures

Supplementary Tables

Supplementary References

Supplementary Methods

KH-9 DEM generation and SRTM/Copernicus DEM datasets

In this study, we collected approximately 200 KH-9 images, consisting of 145 pairs of stereo images, representing nearly all available data (excluding those affected by heavy cloud coverage) for the glacierized areas of the HMA. The data coverage conditions are presented in Fig. S8 and Table S4. To generate KH-9 DEMs, we used the HEXagon IMagery Automated Pipeline (HEXIMAP), an automatic data processing tool developed by Maurer et al. (2015). This tool combines traditional photogrammetry techniques with computer vision concepts (e.g., the structure-from-motion technique). For detailed methodology, please refer to Maurer et al. (2015). The final KH-9 DEMs were generated with geographic information by matching the three-dimensional point cloud to SRTM data based on an integrated approach combing a “spin image” surface registration method with a downhill simplex solver (Maurer et al., 2015), with a spatial resolution of 30 m. Additionally, we obtained the global 1-arcsecond SRTM DEM (acquired in 2000) and Copernicus GLO-30 DEM (acquired for 2011-2015), which is an improved version of the TanDEM-X DEM. Both datasets have a horizontal resolution of approximately 30 m. The entire data processing workflow for this study is outlined in Fig. S9.

DEM differencing and systematic error corrections

Before performing DEM differencing, we first converted the vertical reference frame of the Copernicus DEM from EGM2008 to EGM96, which is consistent with the reference system used for the SRTM DEM and KH-9 DEM data. Subsequently, a stepwise processing strategy was applied for DEM differencing, consisting of global coarse correction followed by local fine correction. Specifically, the first step involved data preprocessing. Glacier outlines from the Randolph Glacier Inventory (RGI) version 7.0 were used to mask glacierized areas and extract DEMs from ice-free areas (RGI Consortium, 2023). At the same time, a slope-based filter was applied to remove pixels with terrain slope exceeding 45°. The second step involved correcting horizontal displacements between DEMs using a global DEM co-registration method based on slope and aspect information (Nuth and Kääb, 2011). Subsequently, a quadratic polynomial fitting method was applied to remove spatial trend biases. In particular, in the elevation difference map between the KH-9 DEM and the SRTM DEM, stripe biases (characterized by high-frequency undulation) possibly caused by image digitization and scanning were corrected on a pixel-by-pixel basis using the average value of samples along the track and cross-track directions. In the third step, the elevation difference map was divided into regular data tiles (1000 × 1000 pixels), and data co-registration and spatial detrending (using linear fitting) were performed individually for each tile. The local spatial trends mainly resulted from chunk-based stereo matching process. The results of this processing workflow are shown in Figs. S10-S11.

Additionally, we employed the method proposed by Zhou et al. (2018) to remove outliers of elevation changes in high altitude glaciated regions. Specifically, for each elevation difference map, the first step involved normalizing the elevation within the glacierized areas using the following expression:

$$\omega = \left(H_{max} - H_{gla}\right)/(H_{max} - H_{min}) \tag{1}$$

where w is the normalized elevation parameter, H_{max}, H_{min}, and H_{gla} represent the maximum elevation, minimum elevation, and the elevation of a given point within the glacier extent, respectively. The second step involved calculating the potential range of glacier

elevation change (Δh_{range}), which can be expressed as:

$$\Delta h_{range} = \gamma \times \omega^2 \quad (2)$$

where γ represents the theoretical maximum elevation change (usually appeared in the glacier front). Based on prior experience, γ was set to 1.5 times the maximum elevation change. A detailed description of this method can be found in Zhou et al. (2018). It should be noted that, in localized areas affected by glacier surging or the presence of proglacial and supraglacial lakes, actual glacier elevation changes may exceed the estimated range of variation. As a result, some valid observations may be identified as outliers, leading to localized data gaps and possibly biases in the individual glacier mass balance estimates. Furthermore, because the remaining observations in some high-altitude regions tend to be dominated by negative elevation changes after these outliers are removed, the subsequent gap-filling procedure may introduce a slight systematic bias toward negative elevation changes (i.e., glacier thinning). However, the magnitude of this potential bias is difficult to quantify. The final glacier elevation change and statistical information for ice-free areas are presented in Fig. S12-S15.

To further consider temporal difference of data acquisition, we took the 1975-2000 and 2000-2013 for the reference period and performed the temporal standardization for elevation difference maps. For the temporal standardization, we mainly followed the approaches of Mannerfelt et al (2022). Specifically, for each elevation difference map, the elevation changes were adjusted using the ratio of the cumulative glacier mass balance over the reference period ($B_{1975-2000}$) to that over the corresponding actual observation period (B_{t_0-2000}), as expressed in Eq. (3).

$$\left[\frac{dh}{dt}\right]_{1975-2000} = \left[\frac{dh}{dt}\right]_{t_0-2000} \times \frac{B_{1975-2000}}{B_{t_0-2000}} \quad (3)$$

where t_0 is the years of acquisition of the KH-9 data. In this study, the cumulative mass balance was calculated based on the latest mass balance datasets released by Dussaillant et al. (2025). In addition, for the period 2000-2013, we did not perform the above processing due to the fact that the acquisition time of individual TanDEM-X data is unknown. However, we considered the mass balance changes caused by temporal difference when performing the uncertainty assessment, and detailed information is given below.

Penetration depth bias correction

After removing systematic errors associated with affine transformations, the elevation bias caused by the radar penetration depth in glacierized areas must also be addressed, since the Copernicus DEM and SRTM DEM are generated from X-band and C-band radar interferometric data, respectively. To do this, we first collected estimates from relevant studies (primarily based on the DEM differencing and the ICESat trend extrapolation) for X/C-band penetration depths and their difference across the HMA. Detailed information is summarized in Table S5. Since the X-band penetration depth was only estimated in five localized regions such as the Karakoram (2 m) (Round et al., 2017), the western Kunlun (2.1 m) (Li et al., 2021), Tanggula (1.52 m) (Bhattacharya et al., 2021), Puruogangri (1.56 m) (Liu et al., 2019), and Nyainqentanglha mountains (1.84 m) (Zhou et al., 2022) (Table S5), we applied proximity or average values to assign depths to regions lacking specific estimates. For instance, the X-ban d penetration depth in the eastern Pamir, western Pamir and Hindu Kush mountains was set to 2 m, based on estimates from the nearby Karakoram. For the Gangdise glacierized areas, we set the value to 1.56 m, and for the Himalayas, the average X-band penetration depth of 1.92 m

was used. The remaining regions were assigned a penetration depth of 1.54 m. For C-band penetration depth correction, previous studies have typically used the penetration depth difference derived from SRTM X-band and C-band data as a first-order approximation (Gardelle et al., 2012, 2013; Holzer et al., 2015; Zhou et al., 2018). In addition, some studies have estimated the absolute C-band penetration depth by extrapolating trends from the differences between ICESat altimetry data and the SRTM DEM (Kääb et al., 2012, 2015). However, in regions with substantial glacier elevation changes, the penetration depth estimated using this method may deviate by several multiples (Brun et al., 2017; Zhou et al., 2018). Therefore, in this study, the C-band penetration depth was estimated by combining the X-band penetration depth with the reported difference between the X-band and C-band penetration depths (Zhou et al., 2022). The penetration depth difference is based on the average estimates provided by Li et al. (2021) and Fan et al. (2023) for each study sub-region, with further details presented in Table S6.

During the processing for penetration depth correction, we applied C-band penetration depth corrections to the elevation difference maps for the period 1975-2000. For the period 2000-2013, we separately corrected for both the C-band penetration and the penetration depth difference, averaging the corrected results as the final estimates. The performance of the final correction can be seen in Fig. S3. Moreover, the effective sampling situation of the final data (i.e. the distribution of sampling glacier area along the altitude) for two study periods can refer to Figs. S16-S17.

Seasonality correction of glacier mass balance

To minimize the impact of seasonal snowfall on glacier mass balance over an entire hydrological year, seasonality corrections were applied when DEM data were acquired in different seasons. For the period 1975-2000, to align with the SRTM DEM acquisition time (February), we applied a correction factor of 0.15 w.e. per winter month, based on estimates from the North Hemisphere glaciers for the winter-accumulation type (Gardelle et al., 2013). However, no seasonality correction was made for summer-accumulation type glaciers, as their accumulation rates are relatively low. Further details on the seasonality correction can be found in Table S4. For the Copernicus DEM dataset, assuming an intermediate acquisition year (in 2013), we considered the uncertainty in mass balance caused by seasonal variations to be already incorporated in this assumption, and thus no additional correction was performed.

Spatially autocorrelated random error estimation in elevation difference maps

Accurately estimating multi-scale spatial autocorrelation errors in elevation difference maps is essential for a reliable uncertainty assessment of glacier elevation changes (Hugonnet et al., 2021, 2022). In this study, we employed an adaptive method to estimate the spatially correlated variance across multiple scales for each elevation difference map. The empirical variogram ($\gamma_{Z_{dh}}$) is modelled as the sum of k variogram models ($\mathrm{V}(c_k, r_k, d)$), as shown in Equation (4).

$$\gamma_{Z_{dh}}(d) = \sum_k V_k(c_k, r_k, d) \quad (4)$$

where c and r represent the total correlated variances and correlation length, respectively. d is the spatial lag distance. Additionally, we used the spherical function model ($S(c, r, d)$) to estimate the autocorrelation length at each scale (Eq.5). An example is provided in Fig. S11.

$$S(c,r,d) = \begin{cases} c_0 + c_1\left(\frac{3}{2}\frac{d}{r} - \frac{1}{2}\left(\frac{d}{r}\right)^3\right) & if\ 0 < d < r \\ c & if\ d \geq r \end{cases} \quad (5)$$

where $c = c_0 + c_1$, c_0 and c_1 represent the nugget and the sill, respectively.

Glacier-level and region-wide mass balance estimate and uncertainty assessment

For the estimation of individual glacier mass balance, we first addressed data gaps in the elevation difference maps (especially those derived from the KH-9 DEMs) by filling missing values with the average elevation change of the corresponding elevation band (e.g., 50 m). For elevation bands without valid samples, missing values were filled using the average elevation change of the nearest valid elevation band (Gardelle et al., 2013). If valid elevation bands were available on both sides of a gap, the missing value was assigned as the average of the elevation changes from the two adjacent elevation bands. At the glacier level, we integrated the average elevation changes across the maximum glacier extent (A_0) throughout the entire study period with the corresponding glacier area to calculate the volume change (ΔV). Here, A_0 represents the maximum glacier extent during the study period, derived by manually adjusting the glacier inventory outlines according to the corresponding elevation difference maps. By using a volume-to-mass conversion factor of 0.85 (f) (Huss, 2013), we further calculated the glacier-wide specific mass balance rate ($\dot{B}$) using Eq. (6).

$$\dot{B} = f \times \frac{\Delta V}{(A_0 + A_1)/2} \quad (6)$$

where A_1 represents the glacier area around 2013, which was approximated using the RGI v7.0 dataset. Glaciers smaller than 0.5 km^2 were excluded from the mass balance analysis because their estimates are more susceptible to localized residual biases and data gaps. In addition, glaciers located at the boundaries of the DEMs were excluded if less than 80% of the glacier area was covered by original DEM. For the retained glaciers, elevation bands with valid sampling ratios below 10% were treated as empty bands. Glaciers with more than 40% empty elevation bands were also excluded from further analysis. It should be noted that the 40% threshold refers to the proportion of empty elevation bands rather than the proportion of missing pixels within a glacier. Finally, region-wide glacier mass balance (i.e., at the 1°×1° geographic grid, the mountain-level, and the entire HMA scale) was calculated using an area-weighted method. Total glacier mass change for HMA was then estimated based on the total glacierized area, assuming that unmeasured glaciers experienced changes similar to those of the observed glaciers. Glacier mass balance and total mass change were also calculated for the regions defined by the Global Terrestrial Network for Glaciers (GTN-G) (Table S1 and S2).

For the uncertainty assessment of the glacier mass balance, we mainly followed the approaches outlined in Shean et al. (2020), Hugonnet et al (2021) and Mannerfelt et al. (2022). The uncertainty of the spatially averaged glacier elevation change ($\sigma_{\overline{\Delta h}}$) was first estimated at the glacier level using the calculated multi-scale autocorrelation parameters. The calculation process is briefly outlined in Eq.(7) and Eq.(8).

$$\sigma_{\overline{\Delta h}} = \sqrt{\frac{1}{A}\sum_{k=0}^{m} \sigma^2_{\overline{\Delta h_k}}} \quad (7)$$

$$\sigma^2_{\overline{\Delta h_k}} = \int_A [s_k - S(d, s_k, r_k)]dA \quad (8)$$

where A is the mean glacier area over the study period, calculated as $(A_0 + A_1)/2$, and $\sigma^2_{\overline{\Delta h_k}}$

is the variance component associated with different spatial scales.

Furthermore, the uncertainty of glacier volume change ($\sigma_{\Delta V}$) was estimated (Eq. (9)) using the law of error propagation, taking into account the uncertainties associated with penetration depth correction ($\sigma_{\overline{\Delta h}_p}$), spatial interpolation ($\sigma_{\overline{\Delta \mathrm{h}}_\mathrm{i}}$), and temporal standardization ($\sigma_{\overline{\Delta \mathrm{h}}_t}$).

$$\sigma_{\Delta V} = \sqrt{((\sigma_{\overline{\Delta h}} + \sigma_{\overline{\Delta h}_p} + \sigma_{\overline{\Delta h}_i} + \sigma_{\overline{\Delta h}_t}) \times A)^2 + ((\overline{\Delta h} + \overline{\Delta h}_p + \overline{\Delta h}_i + \overline{\Delta h}_t) \times \sigma_A)^2} \quad (9)$$

Where $\overline{\Delta h}$ represents the spatially averaged glacier elevation change, and the subscripts p, i, and t denote the average glacier elevation changes corresponding to the uncertainties arising from penetration depth correction, spatial interpolation, and temporal standardization, respectively. σ_A represents the uncertainty in glacier area, which was estimated by assuming a one-pixel uncertainty (e.g., 30 m) along the glacier boundary. For the uncertainty of the penetration depth correction, we used the standard deviation of existing penetration depth estimates across sub-regions in the HMA to approximate the uncertainty. Specifically, the uncertainties were 0.26 m for the X-band penetration and 0.36 m for the penetration depth difference between the X-band and C-band radar waves. This resulted in a C-band penetration uncertainty of 0.44 m. Moreover, to quantify the uncertainty arising from data hole interpolation, we adopted the method proposed by Mannerfelt et al. (2022). Specifically, interpolation uncertainty was estimated by comparing the original and re-interpolated elevation difference values after artificially masking the originally valid pixels and refilling them using the same interpolation method. Artificial gaps were generated as spatially contiguous patches randomly selected from the originally valid observations, with the total masked area matching the observed proportion of missing data within each elevation band, thereby reproducing the observed gap characteristics. Elevation bands with insufficient or no valid observations were excluded from the masking processing. The normalized median absolute deviation (NMAD) of the differences between the reconstructed and original elevation difference values was used to characterize the interpolation uncertainty. These uncertainties were then propagated from the pixel level to the glacier scale by accounting for the spatial autocorrelation of interpolation errors. Following Mannerfelt et al. (2022), we adopted a correlation length of about 2 km, which was originally estimated for glaciers in the Swiss Alps, as an approximation for HMA. Given the broadly comparable glacier sizes in HMA and the Swiss Alps, we consider this assumption to be reasonable for our study, although the correlation length may vary among regions. For the uncertainty resulting from discrepancies in data acquisition times, we first calculated the average rate of glacier mass balance (i.e., −0.21 m w.e./a) in HMA from 1973 to 2015 using the dataset published by Dussaillant et al. (2025). We then performed direct estimations by multiplying this value by the temporal difference. For instance, for the Copernicus DEM data, we accounted for uncertainties due to a ±2-year time difference, yielding an estimated value of ±0.42 m w.e., which was subsequently converted into the corresponding elevation change uncertainty used in Eq. (9).

The final uncertainty of glacier mass balance was further estimated using Eq. (10), taking into account the impact of the seasonality correction ($\sigma_{\Delta B_s,g}$) on glacier mass balance.

$$\sigma_{\Delta B,g} = \sqrt{(\frac{\sigma_{\Delta V} \times f}{A})^2 + (\frac{\sigma_f \times \Delta V}{A})^2 + \sigma_{\Delta B_s,g}^2} \quad (10)$$

where σ_f represents the uncertainty of the density conversion parameter (σ_f). The uncertainty associated with the seasonality correction was set to 100%, with detailed information provided in Table S4.

For the aggregated mass balance uncertainty estimates ($\sigma_{\Delta B,agg}$) at the geographic grid scale (e.g., 1°×1°) or larger spatial extent (e.g., at the mountain or whole HMA scale), these were calculated using Eq. (11), assuming that glaciers are fully correlated.

$$\sigma_{\Delta B,agg} = \frac{\sum_{i=1}^{n}(\sigma_{\Delta B,g,i} \times A_i)}{A_{agg}} \quad (11)$$

where n and A_{agg} represent the number of glaciers and the total area of glaciers within a given geographic extent, respectively. Finally, we incorporated an additional uncertainty of ±0.02 m w.e./a to account for the impact of exclusion of small glaciers (areas less than 0.5 km², representing 12% of the total glacier area in HMA) on regional-scale glacier mass balance estimates. The relevant results can be seen in Fig. S2 and S18.

ERA5 reanalysis data processing

In this study, we collected ERA5 reanalysis data for the period from 1980 to 2010 across the HMA, which includes three main climatic variables: air temperature, precipitation, snowfall. To analyze intra-decadal climate changes, we calculated the difference in the mean annual air temperature (MAAT) and mean annual precipitation between two sub-periods (for 1980-2000 and 2000-2010). Additionally, we further calculated the differences for the mean summer air temperature and mean annual solid precipitation for each mountain/region within the HMA. The corresponding results are shown in Fig. S6.

Sensitivity analyses of glacier mass balance changes to different climatic factors

We conducted a stepwise analysis of variance (ANOVA) combined with model selection criteria to identify the optimal model for explaining the response variable (the glacier mass balance difference) from a set of candidate predictors. First, we considered all possible non-empty subsets of predictor variables (e.g., liquid precipitation, solid precipitation, annual temperature, and summer temperature) using an exhaustive search approach. A linear model was then fitted using ordinary least squares regression for each subset of predictors.

For each candidate model (i.e., each subset of predictors), we performed an ANOVA on the fitted model. This ANOVA decomposes the variance into components attributable to each predictor, testing the significance of each factor's contribution to the response variable. The resulting F-statistics and p-values were used to assess whether the included predictors, either collectively or individually, significantly explain variation in the response variable.

In addition to ANOVA significance tests, we evaluated each model using several statistical criteria: (1) Akaike information criterion (AIC), a measure of model quality that balances goodness of fit and complexity; (2) Bayesian information criterion (BIC), similar to AIC but with a stronger penalty for complex models; (3) adjusted R^2; and (4) root mean square error (RMSE). These metrics provided a balanced assessment, helping to avoid overfitting and guiding us toward a more parsimonious model.

After computing the ANOVA and these criteria for all the candidate models, we ranked the models primarily according to AIC, as lower AIC values indicate better-fitting models with fewer unnecessary predictors. We also considered BIC, adjusted R^2, and RMSE as complementary metrics. The model with the lowest AIC (and favorable values for the other metrics) was selected as the best-performing model. More Details are given in Table S7-S8.

Once the best model was selected, we used the output of the ANOVA function (which includes the sum of squares, sumSq) to quantify the relative contribution of each predictor. By dividing the sumSq of each predictor by the total sumSq assigned to all predictors in the model,

we calculated the proportion of variance explained by that predictor.

Supplementary Figures

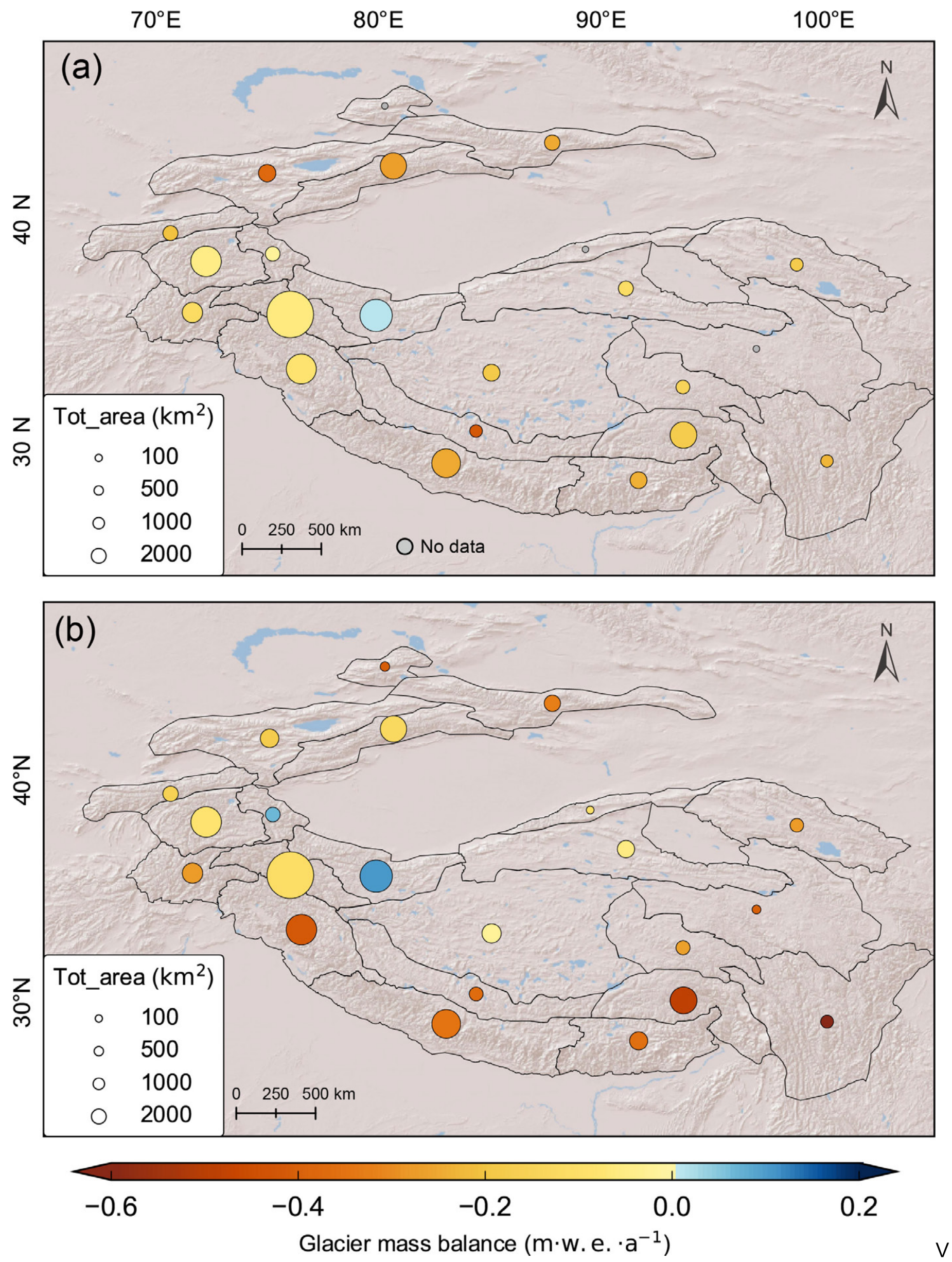

v

Figure S1. Glacier mass balance for each mountain within the HMA for 1975-2000 (a) and 2000-2013 (b). The mountain range boundaries (black lines) are based on the Hindu Kush Himalayan Monitoring and Assessment Programme (HIMAP) project (Bolch et al., 2019).

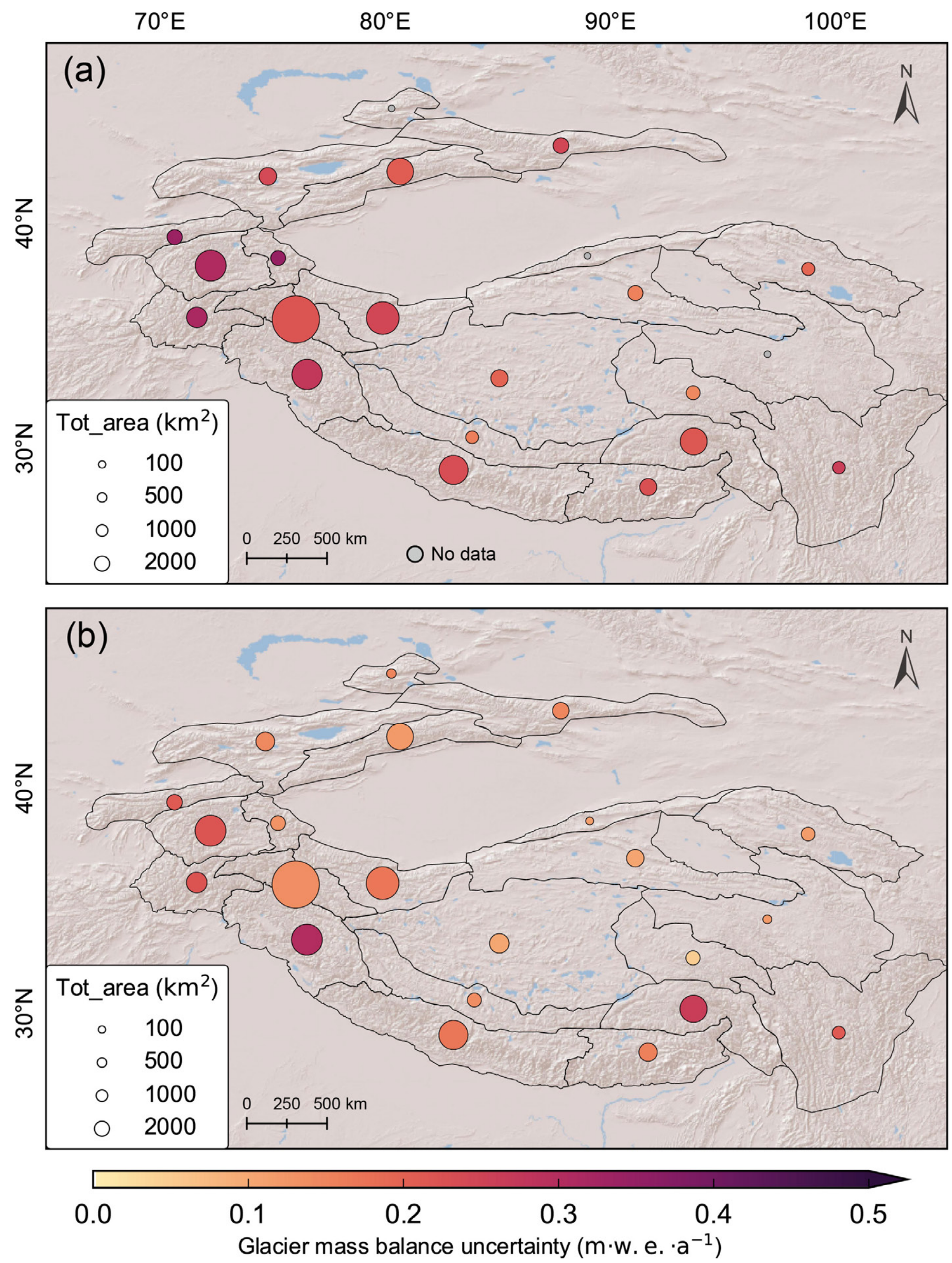

Figure S2. Glacier mass balance uncertainty for each mountain within the HMA for 1975-2000 (a) and 2000-2013 (b).

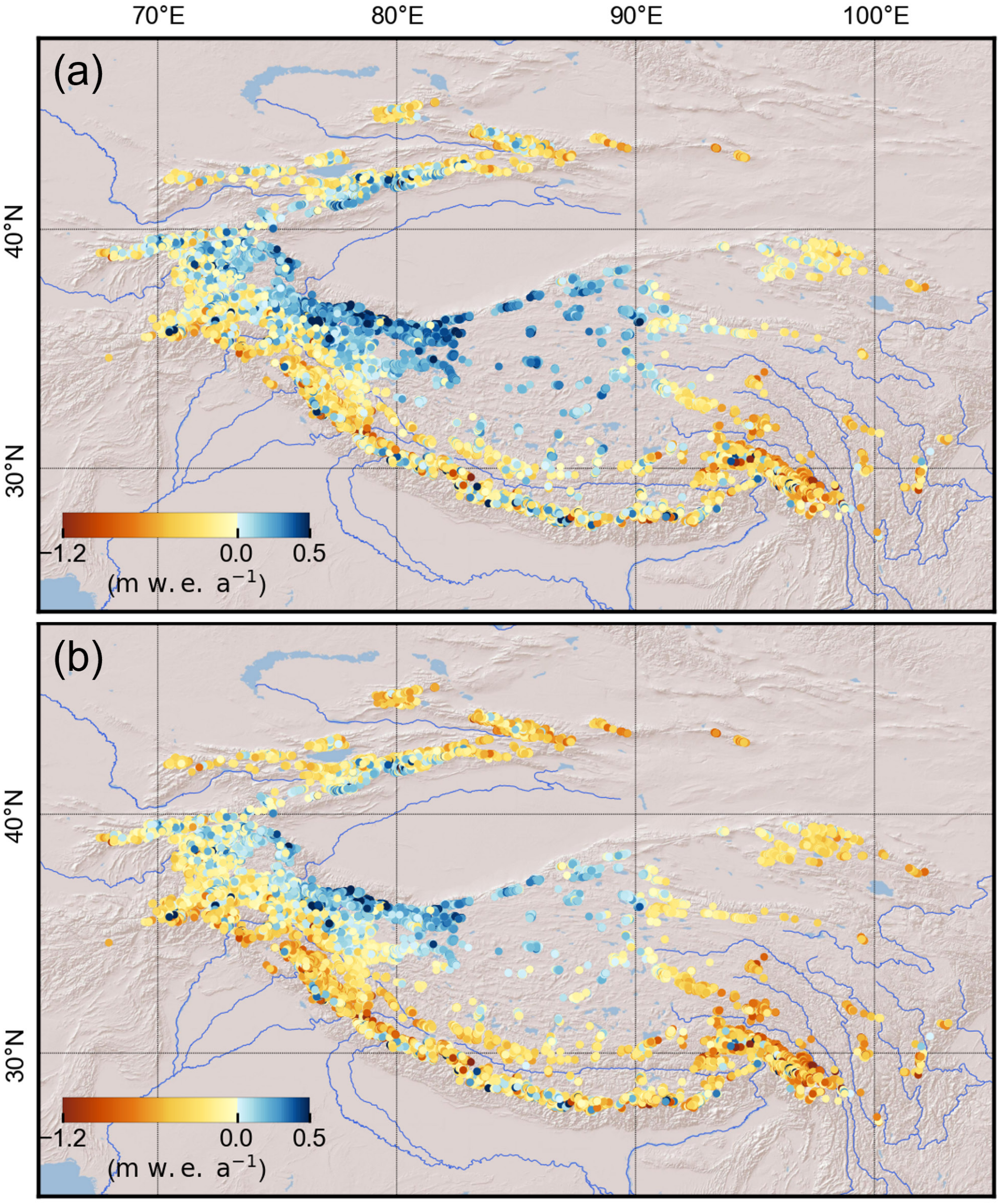

Figure S3. Comparison of glacier-scale mass balance before and after radar penetration depth correction. Panels (a) and (b) show the glacier-level geodetic mass balance for 2000-2013 before and after applying the radar penetration depth correction, respectively.

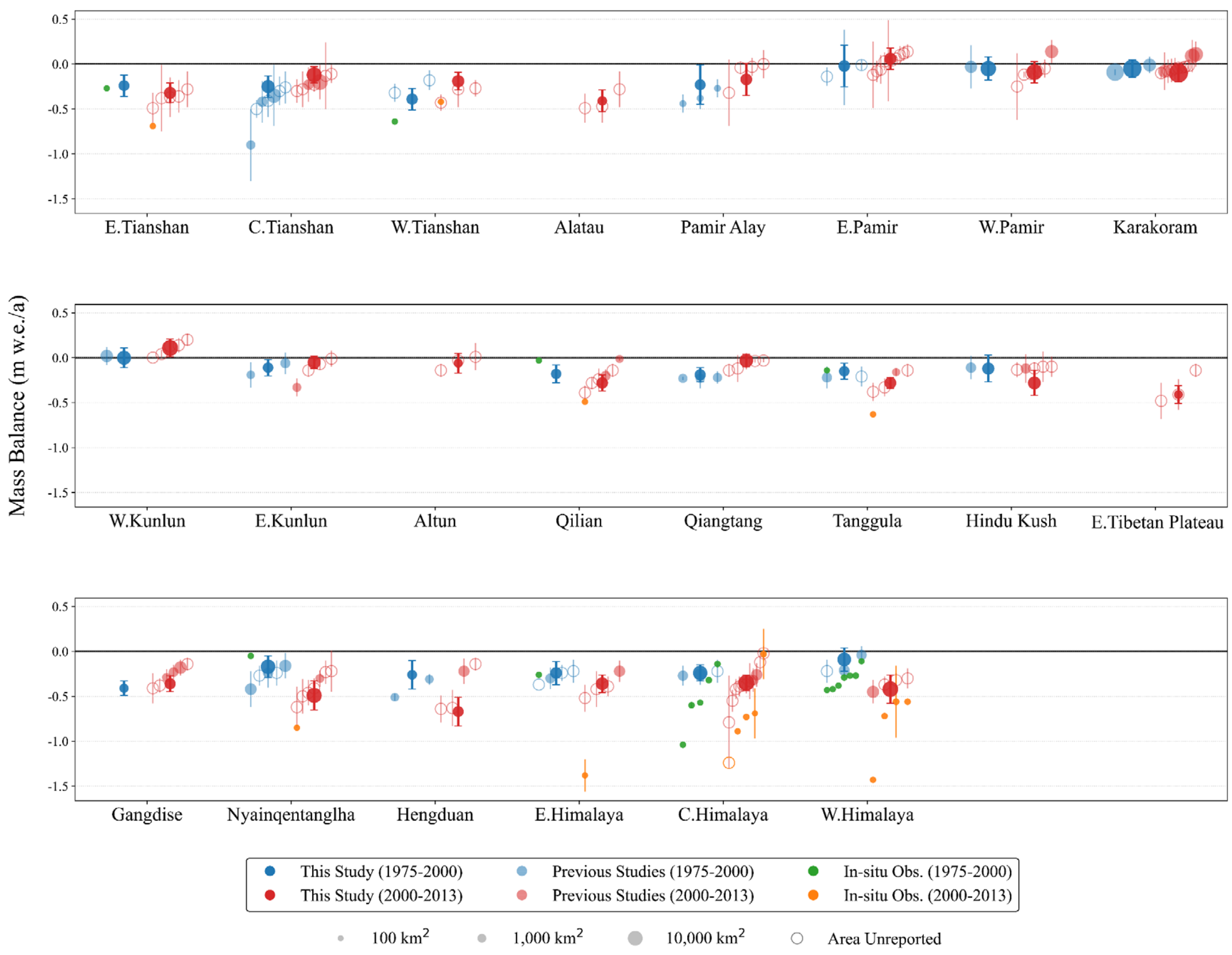

Figure S4. Comparison of region-wide glacier mass balance between this study and previous studies. More detailed results are presented in Table S3.

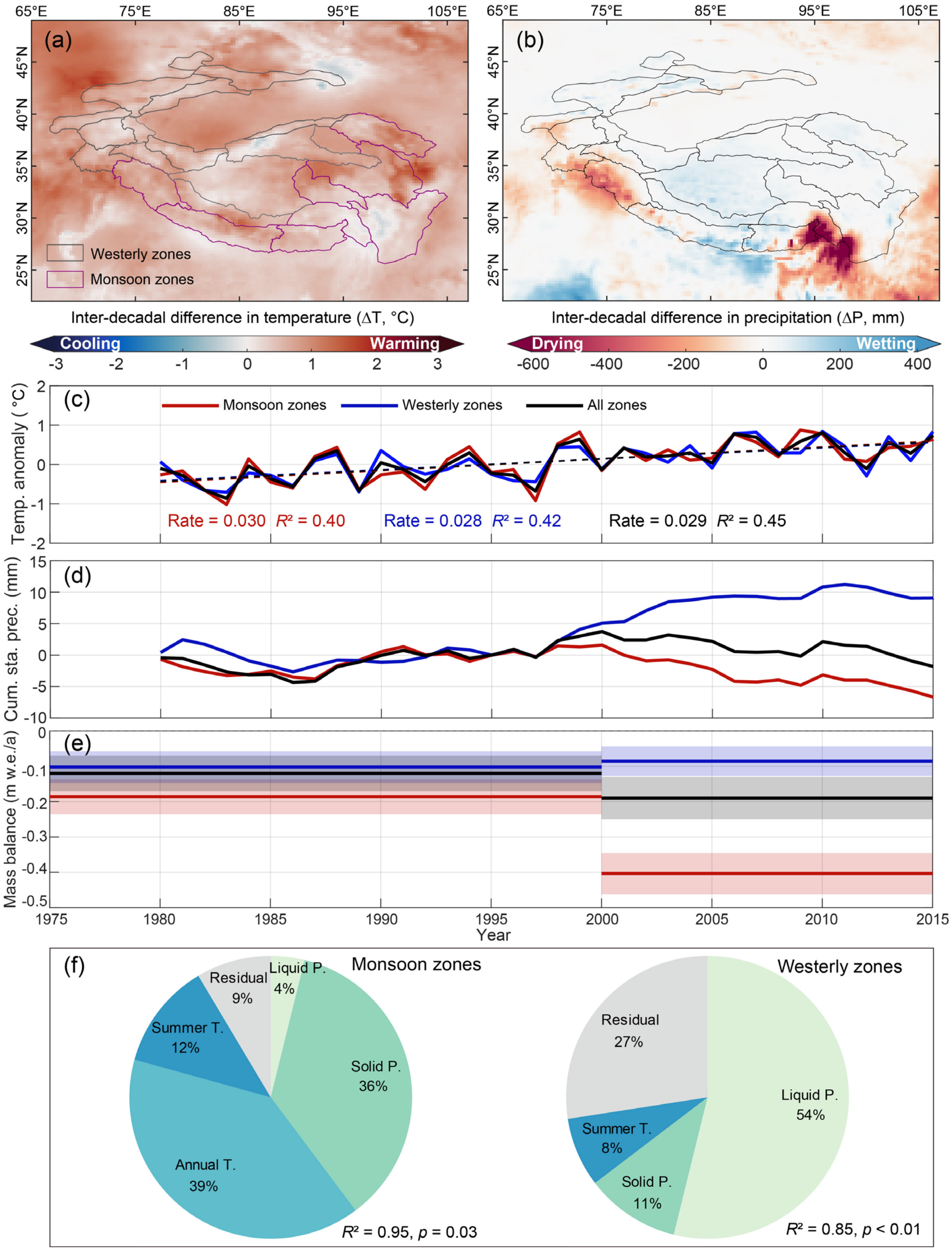

Figure S5. Changes in climatic variables from the 1980s to the 2010s in HMA. Inter-decadal average difference in MAAT (a) and precipitation (b) for 1980-2000 and 2000-2010. (c) the trend of the MAAT anomaly for 1980-2015, (d) Cumulative standardized precipitation for 1980-2015. (e) The difference in glacier mass balance for the two study sub-periods. (f) The contribution of climatic variables to changes in glacier mass balance.

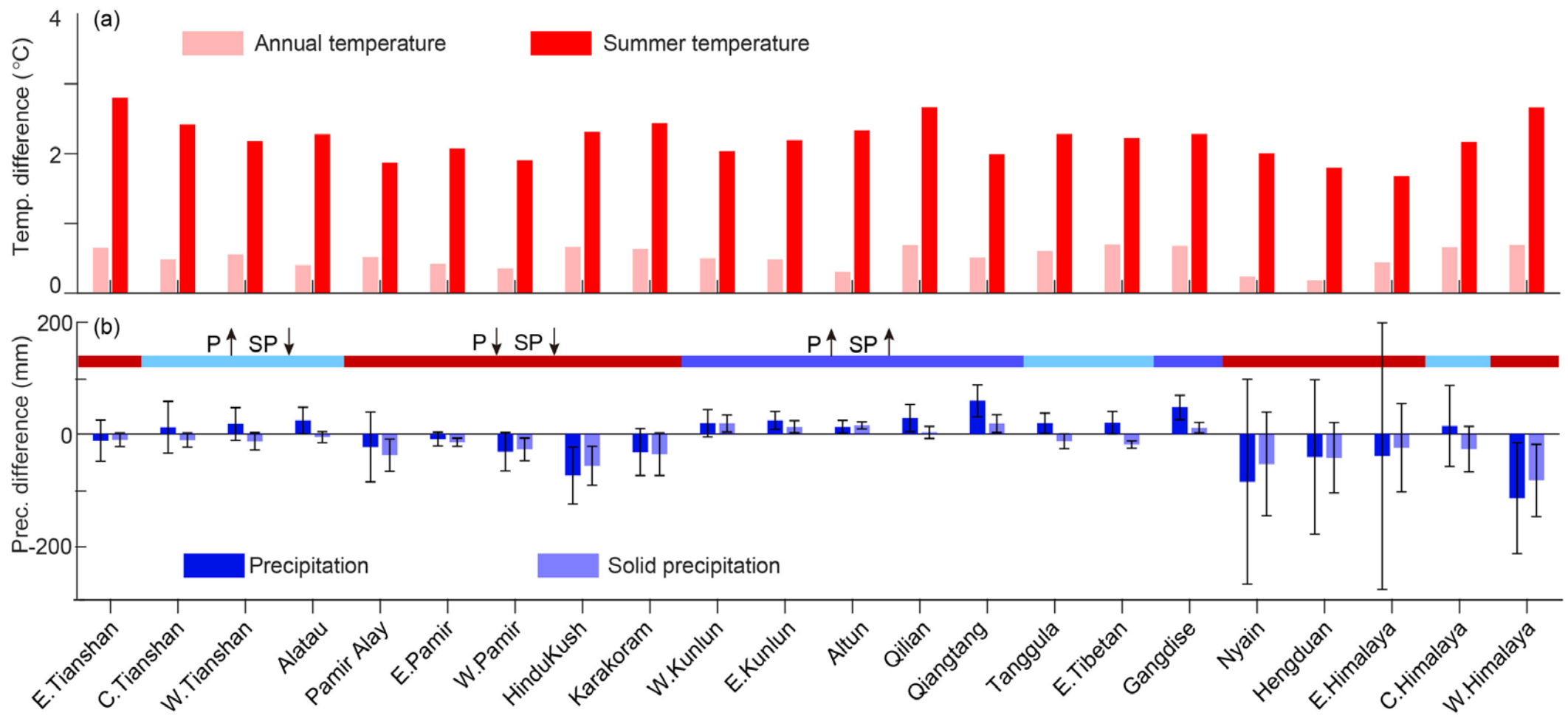

Figure S6. Climatic variable changes for 1980-2010 in the HMA. (a) average difference in MAAT and summer temperature for each sub-region, (b) average difference in AAP and SP for each sub-region.

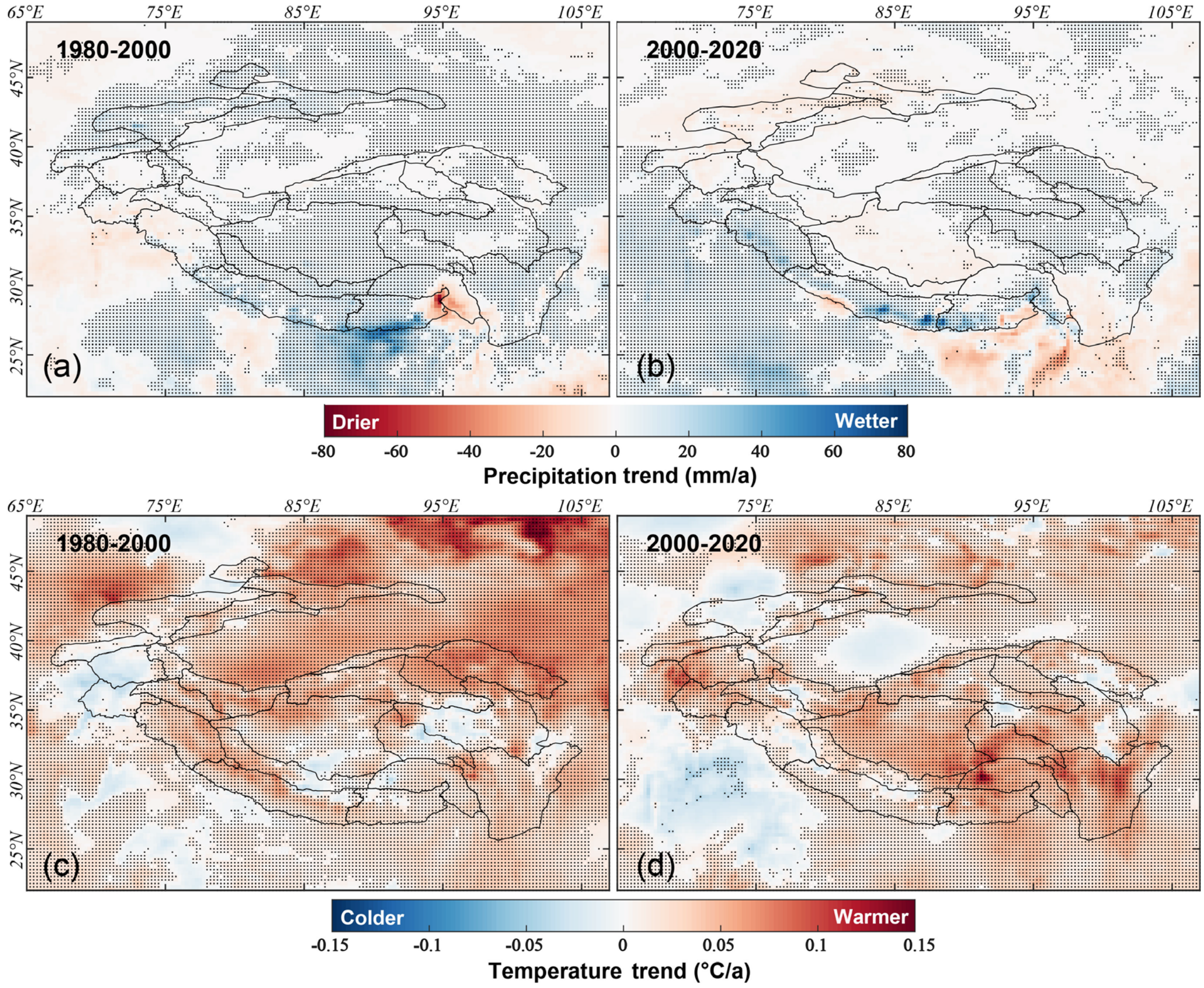

Figure S7. The change trends in climatic variables at two-decades time scales in the HMA. (a) and (b) show the precipitation change trend, and (c) and (d) show the air temperature change trend. Notes that the gray line is the mountain boundary.

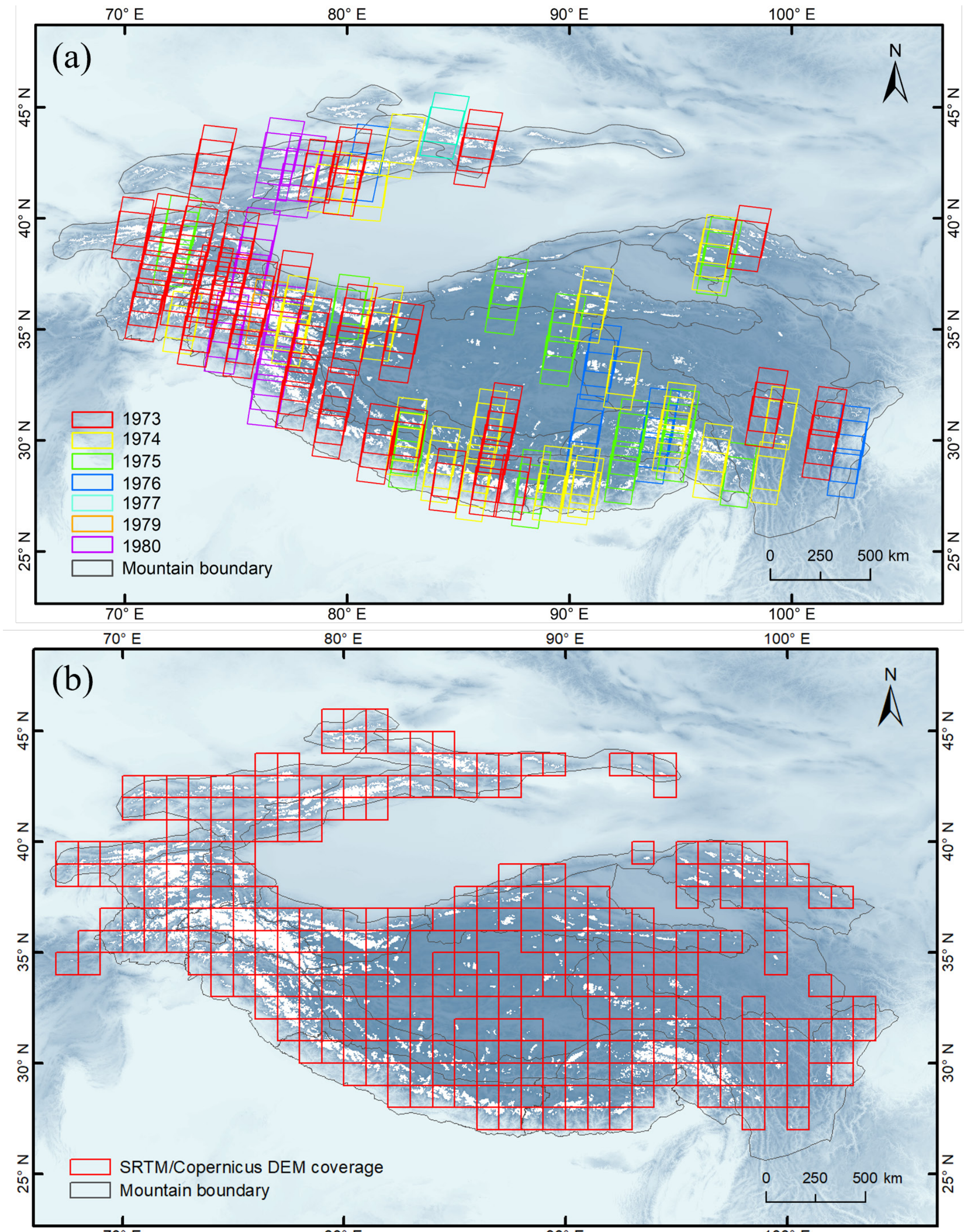

Figure S8. Data coverage condition in HMA. (a) KH-9 images. (b) SRTM GL-1 DEM and Copernicus GLO-30 DEM.

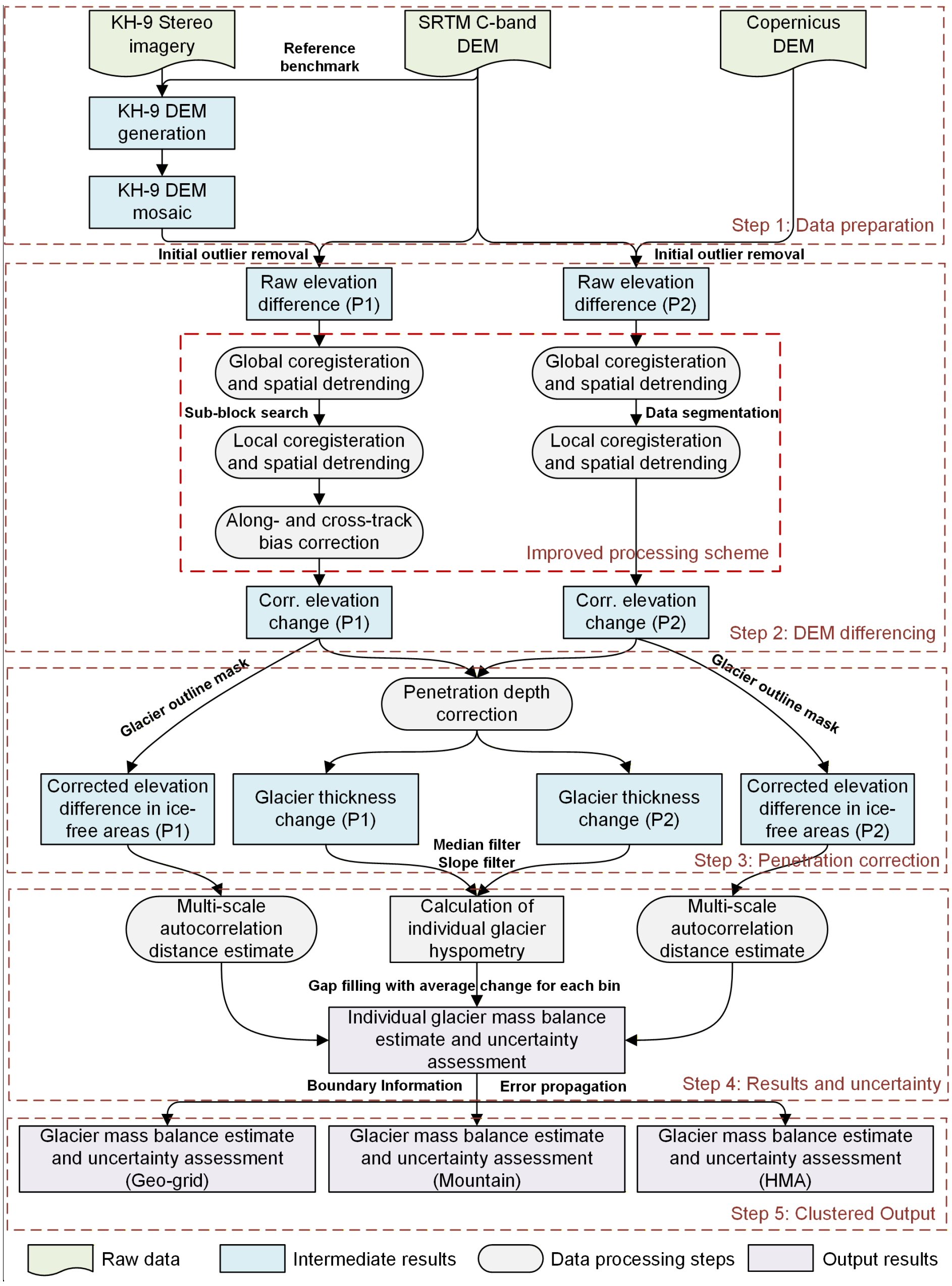

Figure S9. The flowchart of the data processing in this study. P1 and P2 represent the first study period (1975-2000) and the second study period (2000-2013), respectively.

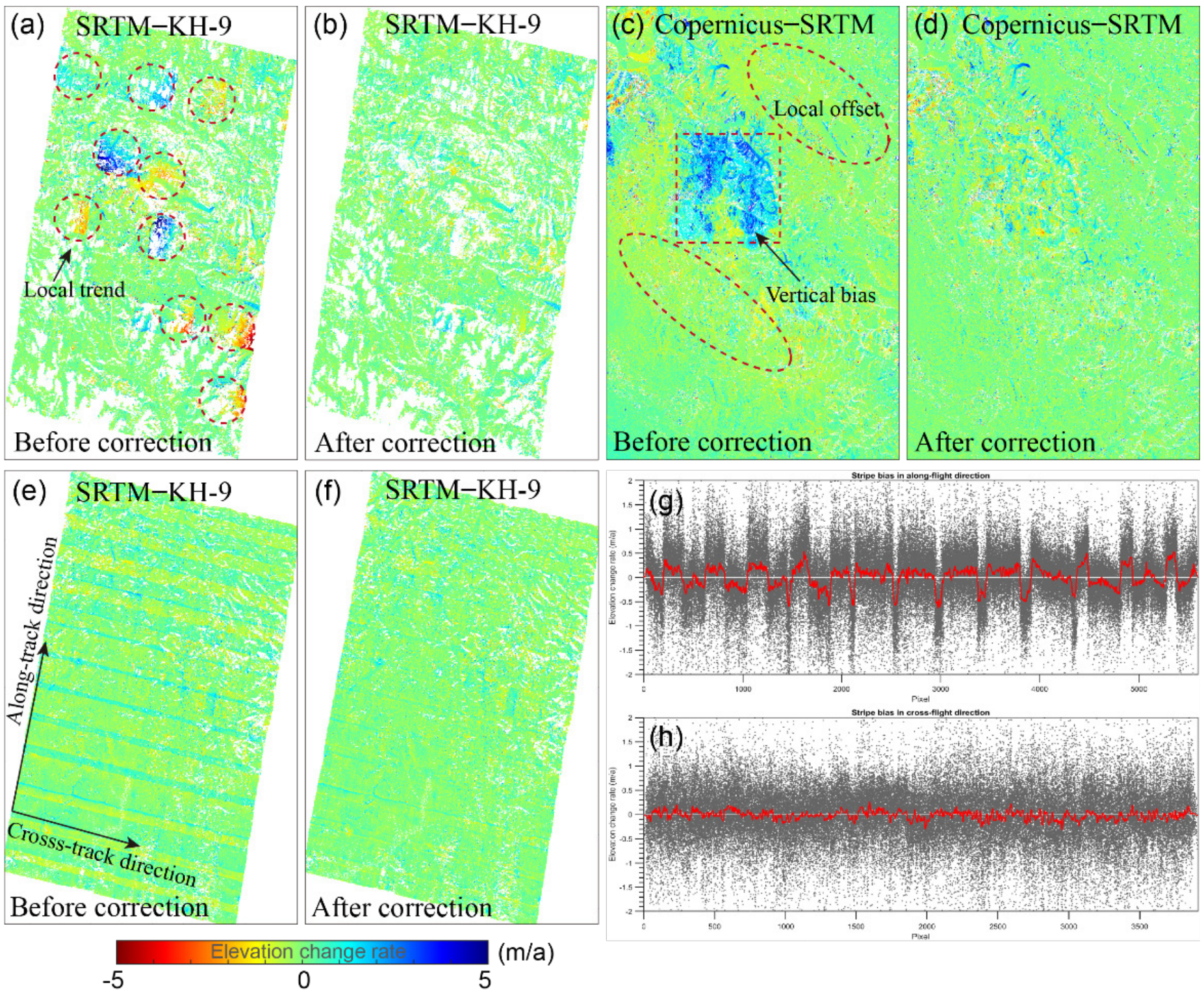

Figure S10. Representative examples of the correction of systematic biases in elevation difference maps. (a) and (b) show the elevation difference map between the KH-9 DEM and the SRTM DEM before and after correcting local spatial trend biases. (c) and (d) show the elevation difference map between the Copernicus DEM and the SRTM DEM before and after correcting local horizontal offsets and lump-shaped vertical biases. (e) and (f) show the mosaiced KH-9 DEM before and after correcting stripe errors. (g) and (h) show the fitted stripe error components in the along-track and cross-track directions, respectively.

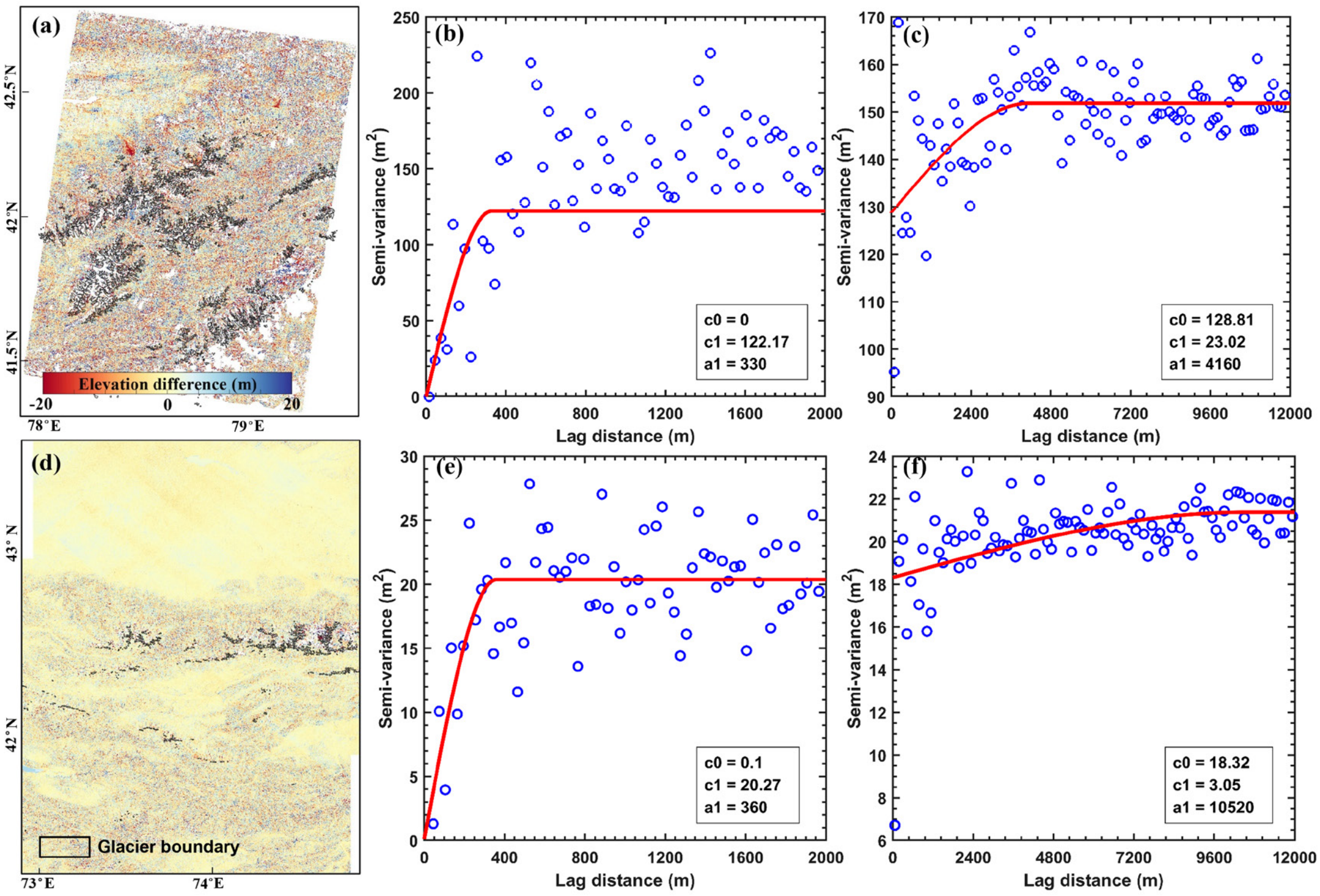

Figure S11. The performance of multi-scale spatially correlated random errors estimation in the ice-free areas. (a), (b) and (c) represent the corrected elevation difference map (SRTM DEM minus KH-9 DEM), short-range and long-range experimental variograms, respectively, in the central Tianshan mountain. (d), (e) and (f) represent the corrected elevation difference map (Copernicus DEM minus SRTM DEM) and its corresponding short-range and long-range variogram.

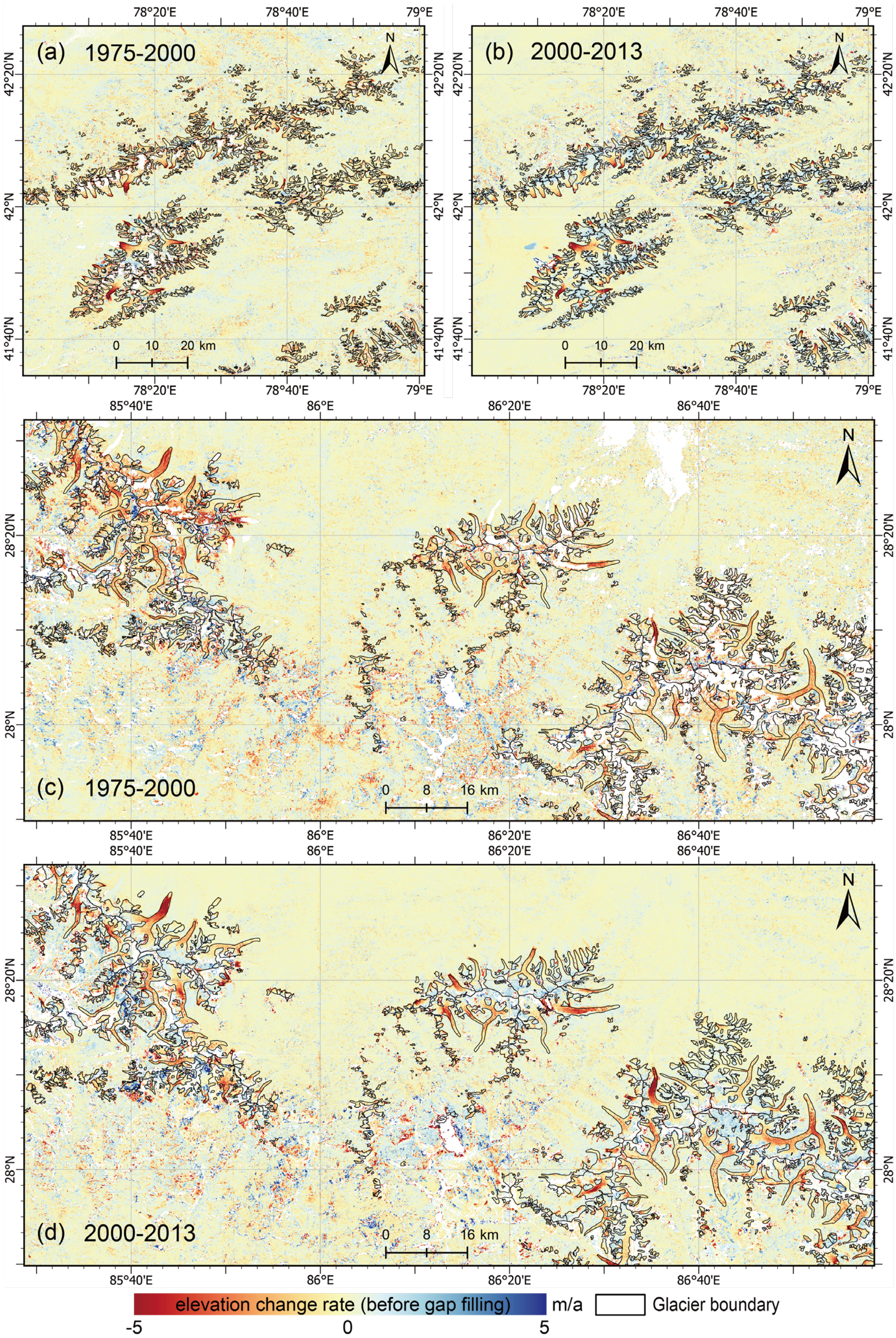

Figure S12. Glacier elevation change maps for two study periods. Taking the western Tianshan mountain ((a) and (b)) and central Himalaya mountain ((c) and (d)) as examples.

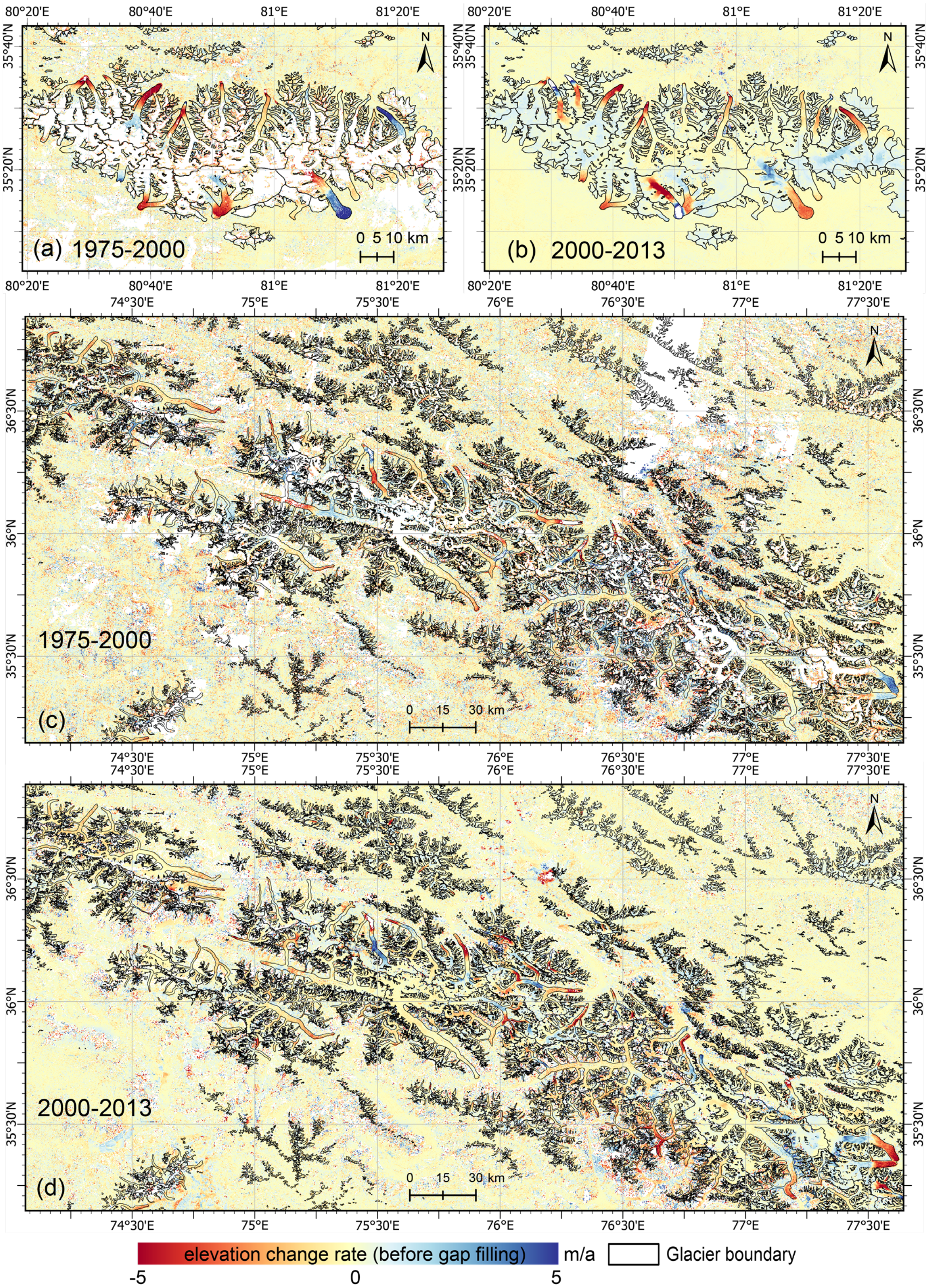

Figure S13. Glacier elevation change maps for two study periods. Taking the western Kunlun mountain ((a) and (b)) and Karakoram mountain ((c) and (d)) as examples.

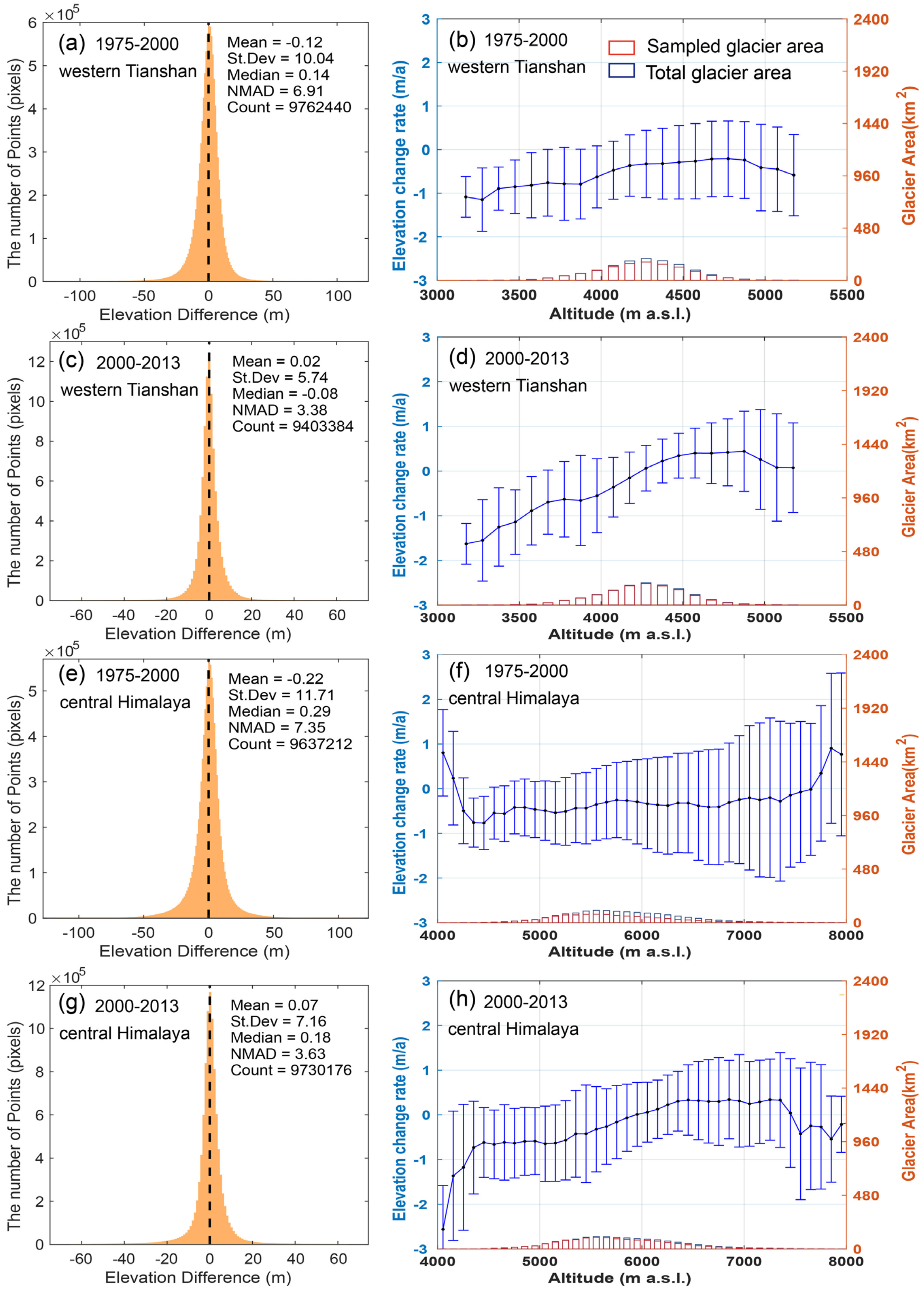

Figure S14. Statistical histogram of elevation differences in stable areas (left column), corresponding to Figure S10, and the relationship between glacier elevation change rates and altitude (right column).

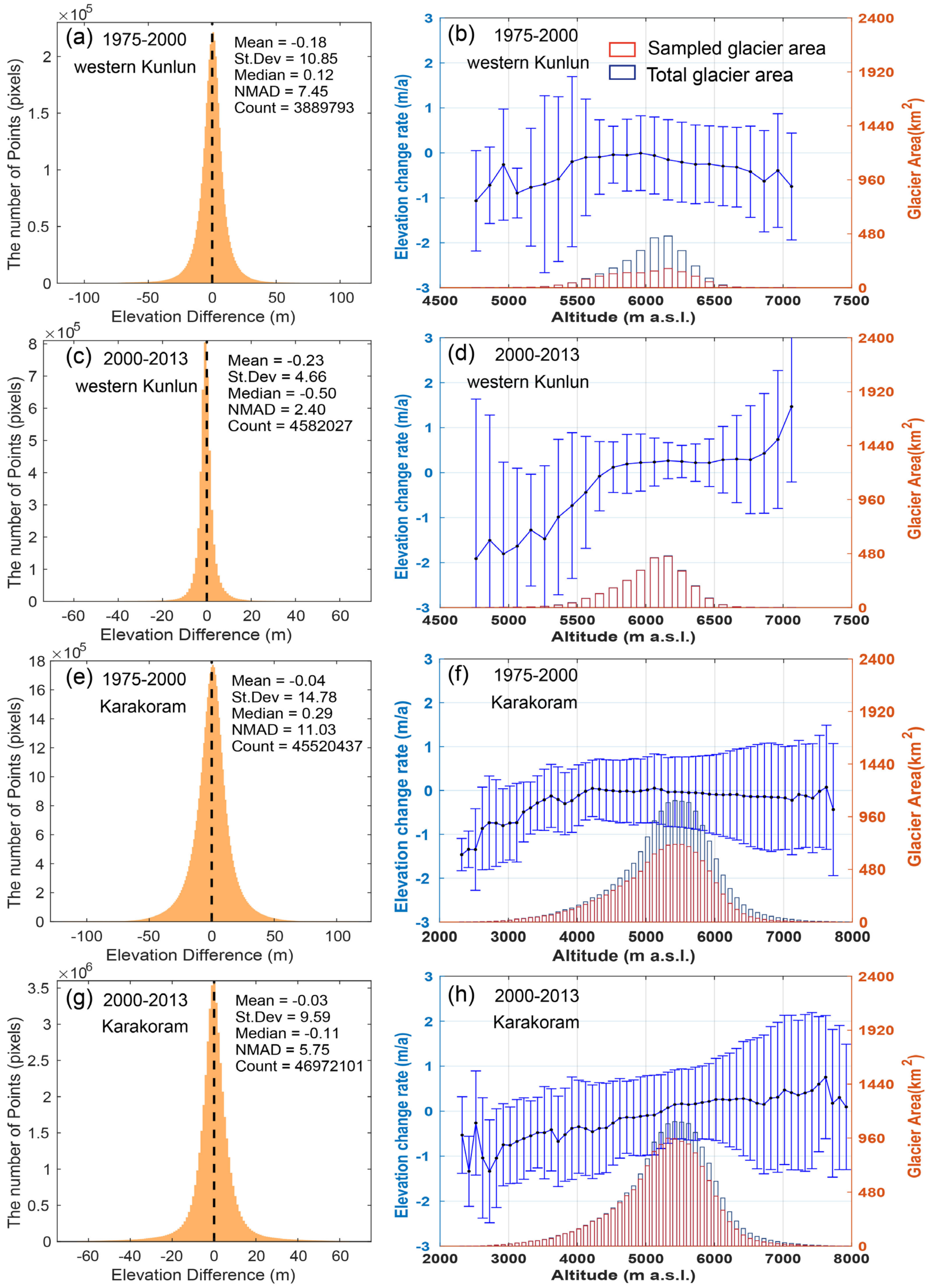

Figure S15. Statistical histogram of elevation differences in stable areas (left column), corresponding to Figure S11, and the relationship between glacier elevation change rates and altitude (right column).

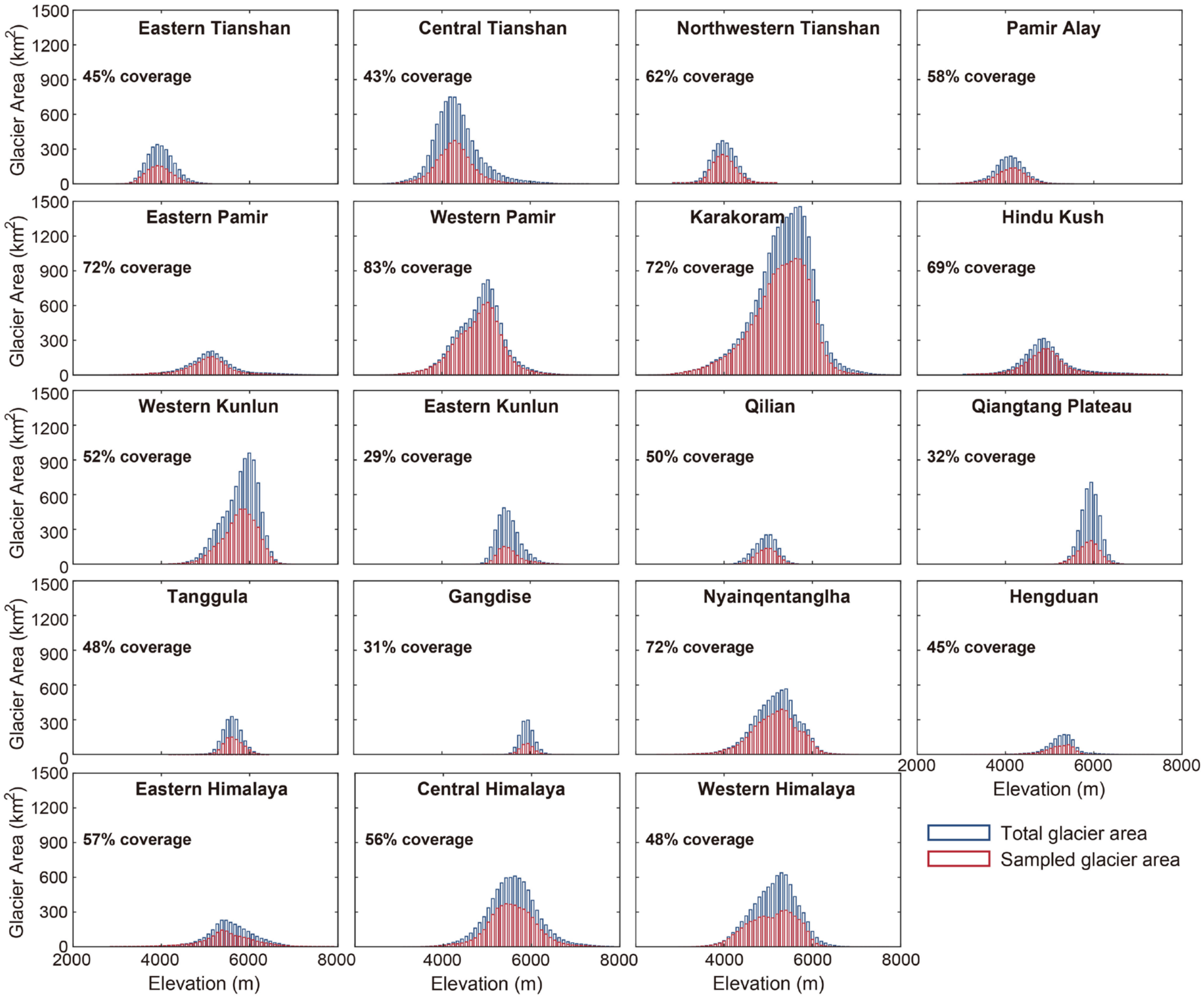

Figure S16. The area distribution of glaciers along the altitude and sampling condition of KH-9 DEM data coverage for each mountain within the HMA.

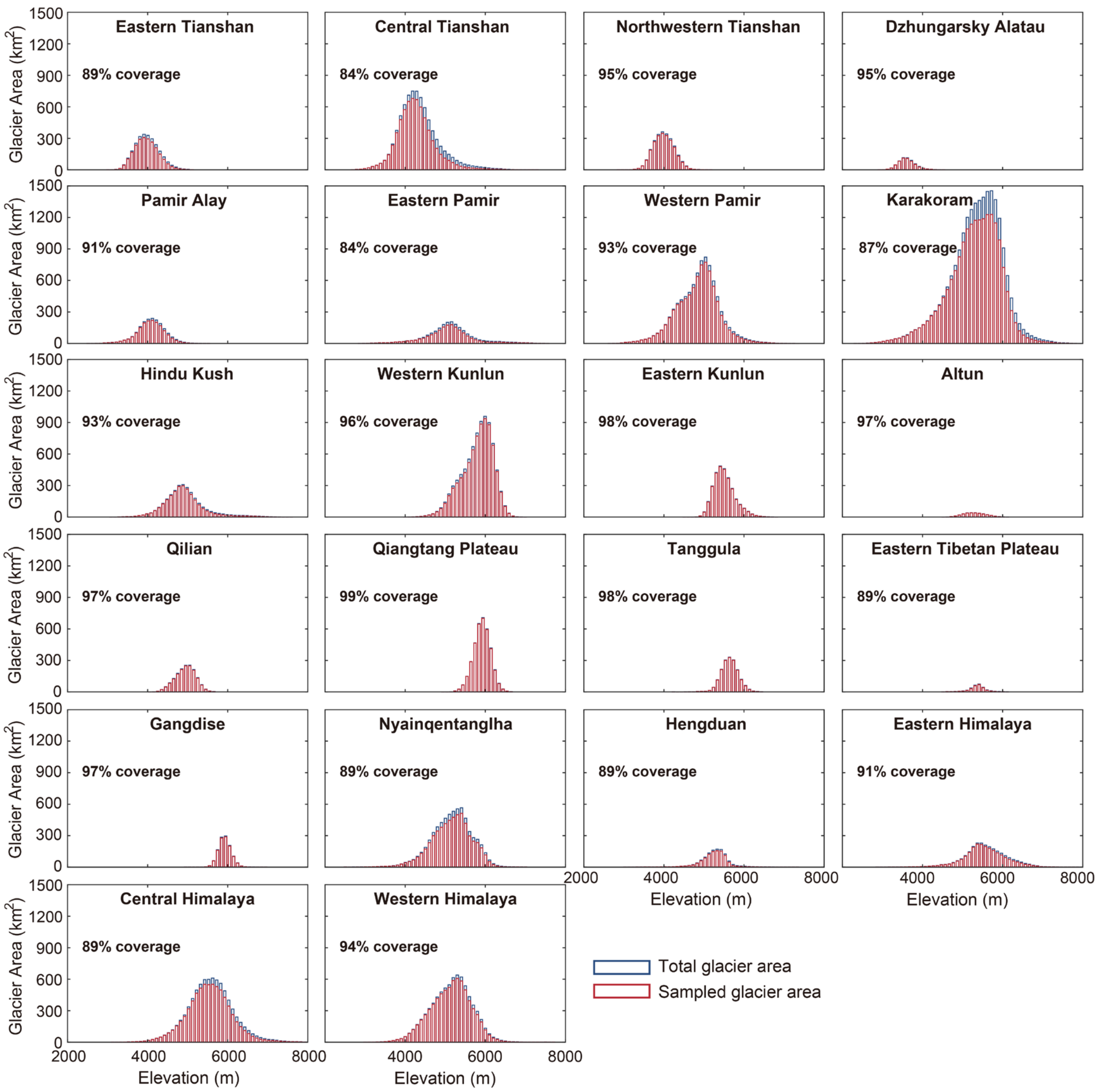

Figure S17. The area distribution of glaciers along the altitude and sampling condition of Copernicus/SRTM DEM data coverage for each mountain within the HMA.

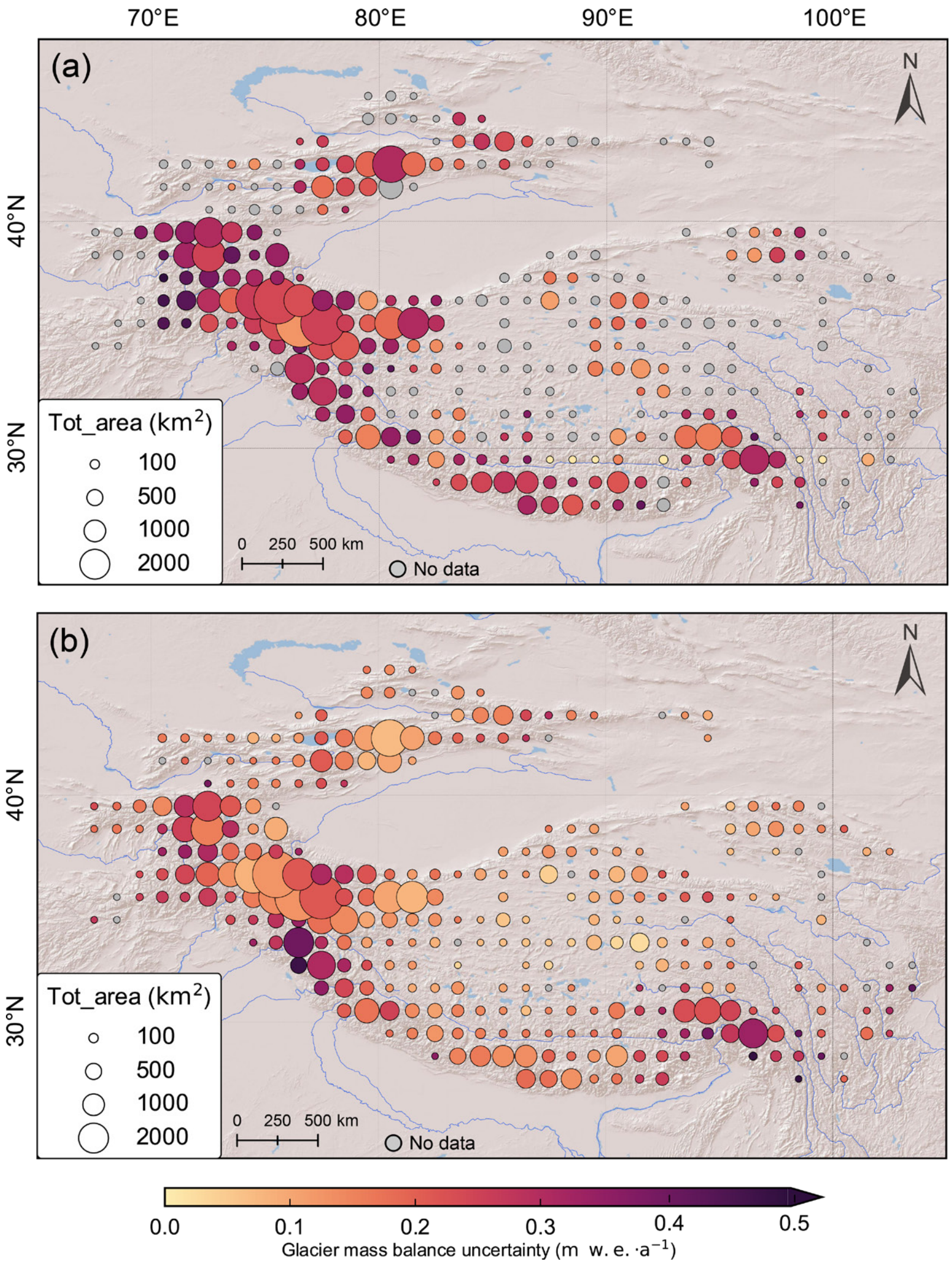

Figure S18. The uncertainty of glacier mass balance at 1° geographic grid scale for 1975-2000 (a) and 2000-2013 (b).

Supplementary Tables

Table S1. The estimated geodetic mass balance for 1975-2000 and 2000-2013 over the HMA. Note that the last column represents the difference in glacier mass balance for the two study periods.

Region	Total area (km^2)	Glacier mass balance (w.e./a)			Glacier state changes[b] (w.e./a)
		1975−2000	2000−2013	2000−2010s[a]	
Eastern Tianshan	2333	−0.25±0.24	−0.33±0.15	−0.36±0.18	Accelerating −0.08
Central Tianshan	7270	−0.27±0.21	−0.13±0.12	−0.11±0.10	Decelerating +0.14
Western Tianshan	2261	−0.38±0.24	−0.18±0.14	−0.26±0.16	Decelerating +0.20
Dzhungarsky Alatau	521	——	−0.41±0.15	−0.47±0.18	——
Pamir Alay	1846	−0.21±0.34	−0.16±0.21	0.00±0.16	Stable +0.05
Eastern Pamir	2201	−0.02±0.34	0.06±0.13	0.10±0.10	Switching +0.08
Western Pamir	8480	−0.05±0.31	−0.09±0.22	−0.05±0.10	Stable −0.04
Karakoram	21475	−0.06±0.22	−0.11±0.14	−0.04±0.06	Stable −0.05
Hindu Kush	3051	−0.12±0.31	−0.28±0.22	−0.10±0.11	Accelerating −0.16
Western Kunlun	8457	0.00±0.24	0.10±0.17	0.20±0.07	Switching +0.10
Eastern Kunlun	2995	−0.11±0.15	−0.05±0.11	−0.01±0.09	Stable +0.06
Altun	295	——	−0.10±0.11	0.01±0.15	——
Qilian	1598	−0.18±0.19	−0.28±0.11	−0.24±0.12	Accelerating −0.10
Qiangtang Plateau	3815	−0.19±0.20	−0.03±0.10	−0.04±0.09	Decelerating +0.16
Tanggula	1841	−0.15±0.14	−0.27±0.08	−0.33±0.10	Accelerating −0.12
Eastern Tibet	312	——	−0.41±0.12	−0.41±0.17	——
Gangdise	1271	−0.42±0.16	−0.37±0.14	−0.41±0.17	Stable +0.05
Nyainqentanglha	7047	−0.18±0.22	−0.49±0.26	−0.50±0.19	Accelerating −0.31
Hengduan	1282	−0.23±0.26	−0.65±0.21	−0.63±0.20	Accelerating −0.42
Eastern Himalaya	2984	−0.24±0.22	−0.36±0.15	−0.39±0.11	Accelerating −0.12
Central Himalaya	8986	−0.25±0.23	−0.35±0.17	−0.39±0.11	Accelerating −0.10
Western Himalaya	8118	−0.09±0.27	−0.42±0.30	−0.30±0.11	Accelerating −0.33
The whole HMA	98439	−0.13±0.23	−0.20±0.17	−0.22±0.05	Accelerating −0.07

a. This column shows the geodetic glacier mass balance reported by Hugonnet et al. (2021), derived from time-series ASTER DEMs.

b. For glacier status variations, we define a continuous mass change (denoted as “sustaining”) when the difference in glacier masss balance between the two periods is within ±0.06 m w.e./a. A deceleration of glacier mass loss is defined when the change from the earlier to the later period exceeds +0.06 m w.e./a (denoted as “decelerating”), while an acceleration of mass loss is defined when the change is less than –0.06 m w.e./a (denoted as “Accelerating”). Additionally, in regions where glacier mass balance shifts from negative to positive, this is defined as a state transition (denoted as “switching”).

Table S2. Glacier mass balance and mass changes in the GTN-G regions during the two study periods.

Region	Glacier Area (km^2)	1975-2000		2000-2013	
		Glacier mass balance (m w.e./a)	Glacier mass change (Gt/a)	Glacier mass balance (m w.e./a)	Glacier mass change (Gt/a)
Central Asia	50344	−0.12±0.24	−6.04±12.08	−0.12±0.16	−6.04±8.06
South Asia west	33075	−0.06±0.24	−1.98±7.94	−0.20±0.21	−6.62±6.95
South Asia east	16049	−0.21±0.25	−3.37±4.01	−0.43±0.22	−6.90±3.53

Table S3. The comparison of the glacier mass balance results of this study with existing studies over the HMA. Note that the area represents sampled glacier area in different studies.

Mountain	1975-2000				2000-2013			
	Mass balance (w.e./a)	Area (km^2)	Period	Sources	Mass balance (w.e./a)	Area (km^2)	Period	Sources
Eastern Tianshan	**−0.25±0.24**	**1050**	1975-2000	**This study**	**−0.33±0.15**	**2076**	2000-2013	**This study**
	−0.27*[a]	1.95	1975-1996	Wang et al. (2012)	−0.28±0.20	N/P	2000-2016	Brun et al. (2017)
					−0.37±0.22	N/P	1999-2015	Zhang et al. (2018)
					−0.38±0.37	N/P	2000-2018	Barandun et al. (2021)
					−0.49±0.17	N/P	2000-2018	Shean et al. (2020)
					−0.36±0.18	N/P	2000-2010s	Hugonnet et al. (2021)
					−0.27*	1.95	1996-2010	Wang et al. (2012)
Central Tianshan	**−0.27±0.21**	**3126**	1975-2000	**This study**	**−0.13±0.12**	**6107**	2000-2013	**This study**
	−0.42±0.23	846	1973-2000	Pieczonka et al. (2013)	−0.23±0.19	839.9	1999-2009	Pieczonka et al. (2013)
	−0.35±0.34	5000	1973-2000	Pieczonka et al. (2015)	−0.28±0.20	N/P	2000-2016	Brun et al. (2017)
	−0.90±0.40 (AK[b])	380	1973-1980	Goerlich et al. (2017)	−0.20±0.19	7240	2000-2013	Li et al. (2018)
	−0.30±0.16 (AK)	N/P	1964-1973	Bhattacharya et al. (2021)	−0.23±0.08	N/P	2000-2018	Shean et al. (2020)
	−0.26±0.18 (AK)	N/P	1973-1980	Bhattacharya et al. (2021)	−0.30±0.13 (AK)	N/P	2009-2015	Bhattacharya et al. (2021)
	−0.50±0.10 (AK)	N/P	1980-2002	Bhattacharya et al. (2021)	−0.13±0.37	N/P	2000-2018	Bhattacharya et al. (2021)
	−0.41±0.18 (AK)	N/P	1964-2002	Bhattacharya et al. (2021)	−0.11±0.10	N/P	2000-2010s	Hugonnet et al. (2021)
Western Tianshan	**−0.38±0.24**	**1402**	1975-2000	**This study**	**−0.18±0.14**	**2148**	2000-2013	**This study**
	−0.18±0.11	N/P	1964-1971	Bhattacharya et al. (2021)	−0.28±0.20	N/P	2000-2016	Brun et al. (2017)
	−0.32±0.10	N/P	1971-2000	Bhattacharya et al. (2021)	−0.27±0.09	N/P	2000-2018	Shean et al. (2020)
	−0.64*	2.60	1973-1992	Sun et al. (2015)	−0.43±0.09	N/P	2000-2012	Bhattacharya et al. (2021)
					−0.30±0.37	N/P	2000-2018	Barandun et al. (2021)

					−0.26±0.16	N/P	2000-2010s	Hugonnet et al. (2021)
					−0.42*	2.6	1993-2014	Sun et al. (2015)
Dzhungarsky Alatau	**Null**	**Null**	1975-2000	**This study**	**−0.41±0.15**	**495**	2000-2013	**This study**
					−0.28±0.20	N/P	2000-2016	Brun et al. (2017)
					−0.49±0.16	N/P	2000-2018	Shean et al. (2020)
					−0.47±0.18	N/P	2000-2010s	Hugonnet et al. (2021)
Pamir Alay	**−0.21±0.34**	**1070**	1975-2000	**This study**	**−0.16±0.21**	**1680**	2000-2013	**This study**
	−0.44±0.10 (Ab)	24	1968-2014	Barandun et al. (2015)	−0.04±0.07	1915	2000-2016	Brun et al. (2017)
	−0.38±0.12 (Ab)	24	1975-2015	Denzinger et al. (2021)	−0.03±0.10	N/P	2000-2018	Shean et al. (2020)
	−0.27 (Ab)	24	1968-2020	Kronenberg et al. (2022)	−0.32±0.37	N/P	2000-2018	Barandun et al. (2021)
					0.00±0.16	N/P	2000-2010s	Hugonnet et al. (2021)
Eastern Pamir	**−0.02±0.34**	**1585**	1975-2000	**This study**	**0.06±0.13**	**1849**	2000-2013	**This study**
	−0.04±0.42	274.3	1973-2000	Holzer et al. (2015)	0.04±0.45 (MA)	272.7	1999-2009	Holzer et al. (2015)
	−0.14±0.10	N/P	1967-1973	Bhattacharya et al. (2021)	−0.07±0.53 (MA)	N/P	2009-2013	Holzer et al. (2015)
	−0.01±0.06	N/P	1973-2001	Bhattacharya et al. (2021)	0.14±0.08	N/P	2000-2016	Brun et al. (2017)
					0.04±0.06	N/P	2000-2018	Shean et al. (2020)
					0.03±0.10 (MA)	N/P	2001-2009	Bhattacharya et al. (2021)
					−0.08±0.12 (MA)	N/P	2009-2013	Bhattacharya et al. (2021)
					−0.06±0.16	N/P	2000-2009	Lv et al. (2020)
					0.06±0.04	N/P	2010-2015	Lv et al. (2020)
					0.12±0.08	N/P	2000-2014	Lin et al. (2017)
					−0.12±0.37	N/P	2000-2018	Barandun et al. (2021)
					0.10±0.10	N/P	2000-2010s	Hugonnet et al. (2021)
Western Pamir	**−0.05±0.31**	**7038**	1975-2000	**This study**	**−0.09±0.22**	**7886**	**2000-2013**	**This study**
	−0.03±0.24	2120	1975-2000	Zhou et al. (2019)	0.14±0.13	3178	1999-2011	Gardelle et al. (2013)
					−0.12±0.03	N/P	2000-2014	Lin et al. (2017)

					−0.08±0.07	N/P	2000-2016	Brun et al. (2017)
					−0.11±0.04	N/P	2000-2018	Shean et al. (2020)
					−0.25±0.37	N/P	2000-2018	Barandun et al. (2021)
					−0.05±0.10	N/P	2000-2010s	Hugonnet et al. (2021)
Karakoram	**−0.06±0.22**	**15462**	1975-2000	**This study**	**−0.11±0.14**	**18683**	**2000-2013**	**This study**
	−0.09±0.03	12366	1975-2000	Zhou et al. (2017)	0.11±0.14 (EK)	5328	1999-2010	Gardelle et al. (2013)
	−0.01±0.09	2868	1975-2000	Bolch et al. (2017)	0.09±0.18 (WK)	5434	1999-2008	Gardelle et al. (2013)
					−0.08±0.21	2868	1999-2009	Bolch et al. (2017)
					−0.03±0.07	17734	2000-2016	Brun et al. (2017)
					−0.05±0.12 (EK)	N/P	2000-2009	Kumar et al. (2019)
					−0.07±0.13 (EK)	N/P	2009-2014	Kumar et al. (2019)
					−0.08±0.13 (EK)	N/P	2000-2014	Kumar et al. (2019)
					−0.10±0.06 (EK)	N/P	2000-2014	Lin et al. (2017)
					−0.02±0.06 (WK)	N/P	2000-2014	Lin et al. (2017)
					−0.04±0.04	N/P	2000-2018	Shean et al. (2020)
					−0.04±0.06	N/P	2000-2010s	Hugonnet et al. (2021)
Hindu Kush	**−0.12±0.31**	**2105**	1975-2000	**This study**	**−0.28±0.22**	**2837**	2000-2013	**This study**
	−0.11±0.13	841	1975-2000	Zhou et al. (2018)	−0.12±0.16	793	1999-2008	Gardelle et al. (2013)
					−0.13±0.08	N/P	2000-2014	Lin et al. (2017)
					−0.12±0.07	N/P	2000-2016	Brun et al. (2017)
					−0.10±0.17	N/P	2000-2018	Shean et al. (2020)
					−0.10±0.11	N/P	2000-2010s	Hugonnet et al. (2021)
Western Kunlun	**0.00±0.24**	**4398**	1975-2000	**This study**	**0.10±0.17**	**8119**	2000-2013	**This study**
	0.02±0.10	2355	1975-2000	Zhou et al. (2018)	0.13±0.06	N/P	2000-2014	Lin et al. (2017)
					0.14±0.08	N/P	2000-2016	Brun et al. (2017)
					0.00±0.00	N/P	2000-2018	Zhang et al. (2020)

					0.04±0.05<br>0.20±0.07	N/P<br>N/P	2000-2018<br>2000-2010s	Shean et al. (2020)<br>Hugonnet et al. (2021)
Eastern Kunlun	**−0.11±0.15**<br>−0.06±0.12 (Mz)<br>−0.19±0.14 (ML)	**869**<br>642<br>189	1975-2000<br>1975-2000<br>1970-2000	**This study**<br>Zhou et al. (2018)<br>Zhang et al. (2020)	**−0.05±0.11**<br>−0.14±0.07<br>−0.07±0.07<br>−0.33±0.10<br>−0.01±0.09	**2935**<br>N/P<br>N/P<br>284<br>N/P	2000-2013<br>2000-2016<br>2000-2018<br>2000-2012s<br>2000-2010s	**This study**<br>Brun et al. (2017)<br>Shean et al. (2020)<br>Ke et al. (2022)<br>Hugonnet et al. (2021)
Altun	**Null**	**N/P**	1975-2000	**This study**	**−0.10±0.11**<br>−0.14±0.07<br>−0.03±0.08<br>0.01±0.15	**286**<br>N/P<br>N/P<br>N/P	2000-2013<br>2000-2016<br>2000-2018<br>2000-2010s	**This study**<br>Brun et al. (2017)<br>Shean et al. (2020)<br>Hugonnet et al. (2021)
Qilian	**−0.18±0.19**<br>−0.03*	**799**<br>2.53	1975-2000<br>1961-1995	**This study**<br>Zhang et al. (2022)	**−0.28±0.11**<br>−0.49*<br>−0.14±0.07<br>−0.01±0.02 (No.12)<br>−0.19±0.07<br>−0.28±0.07<br>−0.39±0.08<br>−0.24±0.12	**1550**<br>2.53<br>N/P<br>134.9<br>N/P<br>N/P<br>545<br>N/P	2000-2013<br>1995-2020<br>2000-2016<br>2000-2015<br>2000-2013<br>2000-2018<br>2000-2012s<br>2000-2010s	**This study**<br>Zhang et al. (2022)<br>Brun et al. (2017)<br>Li et al. (2023)<br>Sun et al. (2018)<br>Shean et al. (2020)<br>Ke et al. (2022)<br>Hugonnet et al. (2021)
Qiangtang	**−0.19±0.20**<br>−0.23±0.02 (PIC)<br>−0.23±0.11 (PIC)<br>−0.22±0.07 (PIC)	**1220**<br>387<br>N/P<br>N/P	1975-2000<br>1975-2000<br>1969-1975<br>1975-2000	**This study**<br>Ren et al. (2022)<br>Bhattacharya et al. (2021)<br>Bhattacharya et al. (2021)	**−0.03±0.10**<br>−0.04±0.02 (PIC)<br>−0.14±0.07<br>−0.12±0.15<br>−0.03±0.02 (PIC)<br>−0.04±0.09	**3777**<br>N/P<br>N/P<br>N/P<br>N/P<br>N/P	2000-2013<br>2000-2012<br>2000-2016<br>2000-2018<br>2000-2012<br>2000-2010s	**This study**<br>Liu et al. (2016)<br>Brun et al. (2017)<br>Shean et al. (2020)<br>Bhattacharya et al. (2021)<br>Hugonnet et al. (2021)

Tanggula	**−0.15±0.14**	**884**	1975-2000	**This study**	**−0.27±0.08**	**1804**	2000-2013	**This study**
	−0.22±0.12 (GIC)	720	1975-2000	Zhou et al. (2018)	−0.14±0.07	N/P	2000-2016	Brun et al. (2017)
	−0.21±0.11 (GIC)	N/P	1976-2000	Chen et al. (2021)	−0.38±0.10	N/P	2000-2018	Shean et al. (2020)
	−0.14*	1.60	1955-2008	Wei et al. (2025)	−0.63*	1.60	2005-2023	Wei et al. (2025)
					−0.16±0.04 (Bu)	143	2000-2012	Zhou et al. (2022)
					−0.33±0.10	N/P	2000-2010s	Hugonnet et al. (2021)
Eastern Tibet	**Null**	**N/P**	1975-2000	**This study**	**−0.41±0.12**	**278**	2000-2013	**This study**
					−0.14±0.07	N/P	2000-2016	Brun et al. (2017)
					−0.48±0.20	N/P	2000-2018	Shean et al. (2020)
					−0.41±0.17	N/P	2000-2010s	Hugonnet et al. (2021)
Gangdise	**−0.42±0.16**	**394**	1975-2000	**This study**	**−0.37±0.14**	**1233**	2000-2013	**This study**
					−0.14±0.07	N/P	2000-2016	Brun et al. (2017)
					−0.38±0.08	N/P	2000-2018	Shean et al. (2020)
					−0.29±0.09 (EG)	354	2000-2012s	Ke et al. (2022)
					−0.23±0.08 (CG)	523	2000-2012s	Ke et al. (2022)
					−0.18±0.09 (WG)	2327	2000-2012s	Ke et al. (2022)
					−0.41±0.17	N/P	2000-2010s	Hugonnet et al. (2021)
Nyainqentanglha	**−0.18±0.22**	**5073**	1975-2000	**This study**	**−0.49±0.26**	**6271**	2000-2013	**This study**
	−0.16±0.14*	1986	1975-2000	Zhou et al. (2018)	−0.23±0.13	N/P	2000-2013/14	Li and Lin (2017)
	−0.42±0.20 (YG)	1713	1968-2000	Wu et al. (2019)	−0.62±0.23	N/P	2000-2016	Brun et al. (2017)
	−0.24±0.16 (PZ)	788	1980-2000	Wu et al. (2018)	−0.22±0.23	N/P	2000-2013	Ren et al. (2020)
	−0.27±0.11 (WN)	N/P	1968-1976	Bhattacharya et al. (2021)	−0.46±0.14	N/P	2000-2018	Shean et al. (2020)
	−0.24±0.14 (WN)	N/P	1976-2001	Bhattacharya et al. (2021)	−0.41±0.11 (WN)	N/P	2001-2012	Bhattacharya et al. (2021)
	−0.05*	2.51	1980-2004	Mou et al. (2019)	−0.85*	2.51	2004-2015	Mou et al. (2019)
					−0.30±0.09	271	2000-2012s	Ke et al. (2022)
					−0.50±0.19	N/P	2000-2010s	Hugonnet et al. (2021)

Hengduan	**−0.23±0.26**	**577**	1975-2000	**This study**	**−0.65±0.21**	**1141**	2000-2013	**This study**
	−0.31±0.06 (GG)	228	1969-1989	Cao et al. (2019)	−0.22±0.14	1303	1999-2011	Gardelle et al. (2013)
	−0.51±0.04 (GG)	228	1989-2000	Cao et al. (2019)	−0.14±0.07	N/P	2000-2016	Brun et al. (2017)
					−0.64±0.15	N/P	2000-2018	Shean et al. (2020)
					−0.63±0.20	N/P	2000-2010s	Hugonnet et al. (2021)
Eastern Himalaya	**−0.24±0.22**	**1701**	1975-2000	**This study**	**−0.36±0.15**	**2715**	2000-2013	**This study**
	−0.30±0.12(Sikkim)	689	1975-2000	Zhou et al. (2018)	−0.22±0.12 (Bhutan)	1384	1999-2010	Gardelle et al. (2013)
	−0.24±0.09	N/P	1975-2000	King et al. (2019)	−0.42±0.20	N/P	2000-2016	Brun et al. (2017)
	−0.22±0.13	N/P	1975-2000	Maurer et al. (2019)	−0.52±0.15	N/P	2000-2018	Shean et al. (2020)
	−0.37	N/P	1975-2010	Azam et al. (2018)	−0.39±0.11	N/P	2000-2010s	Hugonnet et al. (2021)
	–0.26* (Ch)	5.6	1979-1986	GSI (2001)	–1.38±0.18* (GL)	0.3	2003-2014	Tshering and Fuji. (2016)
Central Himalaya	**−0.25±0.23**	**5032**	1975-2000	**This study**	**−0.35±0.17**	**7998**	2000-2013	**This study**
	−0.27±0.11	806	1975-2000	Zhou et al. (2018)	−0.33±0.20	N/P	2000-2016	Brun et al. (2017)
	−0.26±0.11	2124	1973-2000	King et al. (2019)	−0.37±0.08	N/P	2000-2018	Shean et al. (2020)
	−0.22±0.13	N/P	1975-2000	Maurer et al. (2019)	−0.42±0.11 (Poiqu)	N/P	2004-2018	Bhattacharya et al. (2021)
	−0.24±0.10 (LT)	N/P	1974-2004	Bhattacharya et al. (2021)	−0.37±0.10 (LT)	N/P	2004-2009	Bhattacharya et al. (2021)
	–1.04* (Du)	2.6	1984-1990	GSI (1991)	−0.55±0.12 (LT)	N/P	2009-2015	Bhattacharya et al. (2021)
	–0.32* (Do)	7.0	1992-2000	Dobhal et al. (2008)	−0.12±0.11 (GM)	N/P	2000-2011	Bhattacharya et al. (2021)
	–0.57* (Ka)	1.9	1991-1993	Liu et al. (1996)	−0.02±0.08 (GM)	N/P	2011-2013	Bhattacharya et al. (2021)
	–0.14* (TB)	7.0	1981-1988	Gautam and Mukh. (1992)	−0.26±0.13 (Everest)	1461	1999-2011	Gardelle et al. (2013)
	–0.60±0.03* (RS)	4.6	1998-1999	Fujita et al. (2001b)	−0.32±0.13 (WN)	908	1999-2011	Gardelle et al. (2013)
					−0.79±0.52 (Everest)	N/P	2002-2007	Bolch et al. (2011)
					−0.39±0.11	N/P	2000-2010s	Hugonnet et al. (2021)
					−0.03±0.28* (Me)	5.1	2007-2015	Sherpa et al. (2017)
					−0.69±0.28* (Po)	0.1	2010-2015	Sherpa et al. (2017)
					−1.24* (CN)	N/P	2010-2015	Sherpa et al. (2017)

					–0.73* (Ch)	6.7	2003-2010	Dobhal et al. (2013)
					–0.89* (Ya)	1.6	2011-2012	Yao et al. (2012)
Western Himalaya	**−0.09±0.27**	**3897**	1975-2000	**This study**	**−0.42±0.30**	**7631**	2000-2013	**This study**
	−0.04±0.10 (SL)	776	1975-2000	Zhou et al. (2018)	−0.56±0.40* (CS)	15.5	2002-2014	Azam et al. (2016)
	−0.21±0.08	1095	1975-2000	King et al. (2019)	−1.43* (Ha)	3.2	2000-2012	Vincent et al. (2018)
	−0.22±0.13	N/P	1975-2000	Maurer et al. (2019)	−0.45±0.13 (SL)	2110	1999-2011	Gardelle et al. (2013)
	–0.27* (Ga)	5.2	1974-1983	Raina et al. (1977)	−0.37±0.09	N/P	2000-2016	Brun et al. (2017)
	–0.38* (Go)	2.0	1976-1985	Sangewar and Sid. (2007)	−0.32±0.08	N/P	2000-2018	Shean et al. (2020)
	–0.27* (Ko)	11.9	1983-1984	Kaul (1986)	−0.30 ± 0.11	N/P	2000-2010s	Hugonnet et al. (2021)
	–0.43* (Ne)	1.3	1975-1984	GSI (2001)	–0.56* (NN)	7.8	2005-2010	Yao et al. (2012)
	–0.11* (Ru)	1.1	1979-1981	Srivastava et al. (2001)	–0.72*(Na)	4.6	2000-2015	Koul and Ganjoo (2010)
	–0.42* (SG)	4.9	1981-1991	GSI (1992)				
	–0.29* (Sh)	9.9	1983-1984	Kaul (1986)				

a. * represents field observation results.

b. The capital letters reflect the abbreviations of the glacier names and area names: AK (Ak-Shyirak massif), MA (Muztagh Ata), Ab (Abramov Glacier), EK (Eastern Karakoram), WK (Western Karakoram), Mz (Muztagh), ML (Malan Mountain), PIC (Puruogangri Ice Cap), GIC (Geladandong Ice Cap), Bu (Bujiagangri region), EG (Eastern Gangdise), CG (Central Gangdise), WG (Western Gangdise), WN (Western Nyainqentanglha), YG (Yigong Zangbo basin), PZ (Parlung Zangbo basin), GG (GongGa mountain), LT (Langtang region), GM (Gurla Mandhata), SL (Spiti Lahual), Ch (Changmekhangpu), GL (Gangju La), Du (Dunagiri), Do (Dokriani), Ka (Kangwure), TB (Tipra Bank), RS (Rikha Samba), Me (Mera), Po (Pokalde), CN (Changri Nup), Ch (Chorabari), Ya (Yala), CS (Chhota Shigri), Ha (Hamtah), NN (Naimona'nyi), Na ((Naradu), Ga (Gara), Go (Gor Garang), Ko (Kolahoi), Ne (Neh Nar), Ru (Rulung), SG (Shaune Garang), Sh (Shishram), N/P (Not provided).

Table S4. Information about KH-9 imagery used in this study and seasonal corrections. Notes that each row in the third column represents one stereo image pair.

Region	Acquisition Time of KH-9 image	ID of KH-9 stereo image pairs	Accumulation type of glaciers	Seasonality correction (m w.e.)
Eastern Tianshan	06/08/1973	DZB1206-500092L005001/L006001/L007001	Summer	No
	26/09/1977	DZB1213-500209L004001/L005001		No
Central Tianshan	18/04/1973	DZB1205-500185L006001/L007001/L008001/L009001	Summer	No
	31/07/1973	DZB1206-500069L018001/L019001		No
	19/12/1974	DZB1209-500159L029001/L030001		No
	21/08/1980	DZB1216-500277L022001/L023001		No
Western Tianshan	07/30/1973	DZB1206-500068L008001/L008001/L010001	Winter	0.15
	31/07/1973	DZB1206-500069L018001/L019001		0.15
	21/08/1980	DZB1216-500277L021001/L022001		0.30
	07/09/1980	DZB1216-500324L001001/L002001/L003001		0.30
Dzhungarsky Alatau	Null	Null	Summer	N/A
Pamir Alay	03/08/1973	DZB1206-500080L017001/L018001	Winter	0.15
	22/11/1973	DZB1207-500041L001001/L002001		0.45
	13/07/1975	DZB1210-500134L004001/L005001		No
Eastern Pamir	04/08/1973	DZB1206-500082L016001/L017001/L018001	Winter	0.15
	07/09/1980	DZB1216-500324L007001/L008001/L009001/L010001		0.30
Western Pamir	18/11/1973	DZB1207-500025L005001/L006001	Winter	0.45
	22/11/1973	DZB1207-500041L002001/L003001/L004001/L005001/L006001		0.45
	06/12/1973	DZB1207-500077L003001/L004001/L005001/L006001/L007001		0.45
	13/07/1975	DZB1210-500134L005001/L006001/L007001		No

Karakoram	04/08/1973<br>18/11/1973<br>19/11/1973<br>23/11/1973<br>16/11/1974<br>17/06/1979<br>16/09/1980	DZB1206-500082L019001/L020001/L021001<br>DZB1207-500025L006001/L007001/L008001<br>DZB1207-500030L011001/L012001/L013001<br>DZB1207-500045L006001/L007001/L008001<br>DZB1209-500081L011001/L012001<br>DZB1215-500431L008001/L009001<br>DZB1216-500361L005001/L006001/L007001	Winter	0.15<br>0.45<br>0.45<br>0.45<br>0.45<br>No<br>0.30
Hindu Kush	18/11/1973<br>22/11/1973<br>06/12/1973<br>20/11/1974	DZB1207-500025L008001/L009001/L010001<br>DZB1207-500041L006001/L007001/L008001<br>DZB1207-500077L007001/L008001<br>DZB1209-500092L001001/L002001	Winter	0.45<br>0.45<br>0.45<br>0.45
Western Kunlun	19/11/1973<br>24/11/1973<br>16/11/1974<br>17/11/1974<br>29/12/1975	DZB1207-500030L010001/L011001<br>DZB1207-500047L015001/L016001/L017001<br>DZB1209-500081L010001/L011001<br>DZB1209-500082L008001/L009001/L010001<br>DZB1211-500093L005001/L006001	Winter	0.45<br>0.45<br>0.45<br>0.45<br>0.30
Eastern Kunlun	05/11/1974<br>16/12/1975	DZB1209-500040L006001/L007001/L008001<br>DZB1211-500046L002001/L003001/L004001	Summer	No<br>No
Altun	Null	Null	Summer	N/A
Qilian	02/12/1973<br>06/11/1974<br>16/06/1975	DZB1207-500069L038001/L039001<br>DZB1209-500048L004001/L005001/L006001<br>DZB1210-500029L012001/L013001/L014001	Summer	No<br>No<br>No
Qiangtang Plateau	21/11/1973<br>24/11/1973<br>29/11/1973<br>23/11/1974	DZB1207-500038L001001/L002001/L003001<br>DZB1207-500047L017001/L018001<br>DZB1207-500062L003001/L004001/L005001<br>DZB1209-500101L002001/L003001/L004001	Summer	No<br>No<br>No<br>No

	20/12/1975	DZB1211-500057L025001/L026001/L027001/L028001		No
Tanggula	01/11/1974<br>06/11/1974<br>07/01/1976	DZB1209-500014L025001/L026001<br>DZB1209-500048L016001/L017001<br>DZB1211-500125L005001/L006001/L007001	Summer	No<br>No<br>No
Eastern Tibet	Null	Null	Summer	N/A
Gangdise	21/11/1973<br>24/11/1973<br>30/10/1974<br>04/11/1974	DZB1207-500038L003001/L004001<br>DZB1207-500047L021001/L022001<br>DZB1209-500002L029001/L030001<br>DZB1209-500033L021001/L022001	Summer	No<br>No<br>No<br>No
Nyainqentanglha	06/11/1974<br>11/11/1974<br>21/12/1975<br>04/01/1976<br>07/01/1976	DZB1209-500048L017001/L018001/L019001<br>DZB1209-500063L003001/L004001<br>DZB1211-500061L010001/L011001/L012001<br>DZB1211-500116L002001/L003001/L004001/L005001<br>DZB1211-500125L010001/L011001/L012001	Summer	No<br>No<br>No<br>No<br>No
Hengduan	26/12/1973<br>30/12/1973<br>07/11/1974<br>07/11/1974<br>11/11/1974<br>25/12/1975<br>16/01/1976	DZB1207-500115L007001/L008001/L009001/L010001<br>DZB1207-500127L011001/L012001/L013001<br>DZB1209-500051L008001/L009001<br>DZB1209-500051L012001/L013001<br>DZB1209-500063L002001/L003001<br>DZB1211-500077L014001/L015001<br>DZB1211-500152L009001/L010001/L011001/L012001	Summer	No<br>No<br>No<br>No<br>No<br>No<br>No
Eastern Himalaya	02/01/1974<br>05/11/1974<br>24/11/1974<br>20/12/1975<br>21/12/1975	DZB1207-500136L006001/L007001/L008001<br>DZB1209-500040L019001/L020001/L021001<br>DZB1209-500106L001001/L002001<br>DZB1211-500057L036001/L037001/L038001<br>DZB1211-500061L012001/L013001	Summer	No<br>No<br>No<br>No<br>No

Central Himalaya	11/11/1973	DZB1207-500001L007001/L008001	Summer	No
	16/11/1973	DZB1207-500013L007001/L008001		No
	21/11/1973	DZB1207-500038L005001/L006001/L007001		No
	24/11/1973	DZB1207-500047L022001/L023001/L024001		No
	29/11/1973	DZB1207-500062L010001/L011001/L012001		No
	14/12/1973	DZB1207-500090L012001/L013001		No
	30/10/1974	DZB1209-500002L030001/L031001		No
	04/11/1974	DZB1209-500033L022001/L023001		No
	23/11/1974	DZB1209-500101L006001/L007001/L008001		No
	12/12/1975	DZB1211-500032L004001/L005001/L00601		No
	20/12/1975	DZB1211-500057L036001/L037001/L038001		No
Western Himalaya	19/11/1973	DZB1207-500030L013001/L014001/L015001/L016001	Winter	0.45
	23/11/1973	DZB1207-500045L009001/L010001/L011001		0.45
	16/11/1974	DZB1209-500081L012001/L013001		0.45
	16/09/1980	DZB1216-500361L007001/L008001/L009001/L010001/L011001		0.30
	07/10/1980	DZB1216-500497L001001/L002001/L003001		No

Table S5. The existing penetration depth results for C/X-band radar and their difference in the glacierized areas of the HMA.

Region	X-band penetration depth (m)			Penetration depth difference (m)			C-band penetration depth (m)		
	Data	Results	Sources	Data	Results	Sources	Data	Results	Sources
Eastern Tianshan	Null	Null	Null		2.14 ± 0.16<br>1.96 ± 0.53	Li et al. (2021)<br>Fan et al. (2023)	Null	Null	Null
Central Tianshan	Null	Null	Null		2.80 ± 0.23<br>1.01 ± 0.68<br>1.8	Li et al. (2021)<br>Fan et al. (2023)<br>Ren et al. (2024)	ICESat	2.2±1.2(s/i)	Pieczonka et al. (2015)
Western Tianshan	Null	Null	Null		2.80 ± 0.23<br>1.01 ± 0.57	Li et al. (2021)<br>Fan et al. (2023)	Null	Null	Null
Dzhungarsky Alatau	Null	Null	Null		2.14 ± 0.16<br>1.96 ± 0.53	Li et al. (2021)<br>Fan et al. (2023)	Null	Null	Null
Pamir Alay	Null	Null	Null	SRTM-C DEM, SRTM-X DEM	3.34 ± 0.34<br>2.16 ± 1.17	Li et al. (2021)<br>Fan et al. (2023)	Null	Null	Null
Eastern Pamir	Null	Null	Null		1.14 ± 0.10<br>1.15 ± 0.72	Li et al. (2021)<br>Fan et al. (2023)	ICESat	4.3±0.9(s)<br>1.5±0.9(i)	Holzer et al. (2015)
Western Pamir	TanDEM-X, GNSS data	3−4 (s)	Lambrecht et al. (2018)		1.82<br><br>1.14 ± 0.10<br>1.15 ± 0.72	Zhou et al. (2019)<br>Lin et al. (2019)<br>Li et al. (2021)<br>Fan et al. (2023)	ICESat	5-6	Kääb et al. (2015)
Karakoram	TanDEM-X	~2	Round et al. (2017)		3.4<br>2.6<br>2.25 ± 0.09<br>0.89 ± 0.73	Gardelle et al. (2013)<br>Zhou et al. (2017)<br>Li et al. (2021)	ICESat	5.5 ± 0.3 (s)<br>1.1 ± 0.5 (i)<br>2.4 ± 0.3	Kääb et al. (2015)

						Fan et al. (2023)			
Hindu Kush	Null	Null	Null		1.7<br>1.39 ± 0.31<br>0.69 ± 0.87	Zhou et al (2018)<br>Li et al. (2021)<br>Fan et al. (2023)	ICESat	5.1 ± 0.7 (s)<br>1.7 ± 0.6 (i)<br>2.4 ± 0.4	Kääb et al. (2015)
Western Kunlun	TanDEM-X, SPOT-6	2.10 ± 0.01	Li et al. (2021)		2.9<br>2.50 ± 0.12<br>1.61 ± 0.78	Zhou et al (2018)<br>Li et al. (2021)<br>Fan et al. (2023)	Null	Null	Null
Eastern Kunlun	Null	Null	Null		2.70（MZ）<br>2.72 (XQF）<br>1.66 ± 0.17<br>1.84 ± 0.75	Zhou et al (2018)<br>Zhou et al (2019)<br>Li et al. (2021)<br>Fan et al. (2023)	Null	Null	Null
Altun	Null	Null	Null		1.66 ± 0.17<br>1.84 ± 0.75	Li et al. (2021)<br>Fan et al. (2023)	Null	Null	Null
Qilian	Null	Null	Null		1.47<br>2.92 ± 0.16<br>2.36 ± 0.68	Sun et al. (2017)<br>Li et al. (2021)<br>Fan et al. (2023)	Null	Null	Null
Qiangtang Plateau	TanDEM-X;<br>TanDEM-X, ASTER	0.61 ± 0.06<br>1.56 ± 0.68 m	Liu et al. (2019)<br>Bhattacharya et al. (2021)		2.25 ± 0.09<br>2.17 ± 0.68	Li et al. (2021)<br>Fan et al. (2023)	Null	Null	Null
Tanggula	TanDEM-X, SPOT-6	1.52±0.01(Ge)	Li et al. (2021)		1.90 (Ge)<br>1.80(Bujia)<br>2.25 ± 0.09<br>2.17 ± 0.68	Zhou et al. (2018)<br>Zhou et al. (2022)<br>Li et al. (2021)<br>Fan et al. (2023)	Null	Null	Null
Eastern Tibet	Null	Null	Null		2.04 ± 0.20<br>0.50 ± 0.58	Li et al. (2021)<br>Fan et al. (2023)	Null	Null	Null
Gangdise	Null	Null	Null		2.25 ± 0.09	Li et al. (2021)	Null	Null	Null

					2.17 ± 0.68	Fan et al. (2023)			
Nyainqentanglha	TanDEM-X, Pléiades	3.19±0.89 (s)<br>0.96±1.10 (i)<br>1.84±0.59	Zhou et al. (2022)		1.30 (PL)<br>1.10 (YG)<br>1.20 (WN)<br>2.39 ± 0.18<br>1.13 ± 0.84	Zhou et al. (2018)<br>Li et al. (2021)<br>Fan et al. (2023)	X-band penetration data, SRTM-X/C DEM	4.66±0.92 (s)<br>2.16±1.13 (i)<br>2.98±0.61	Zhou et al. (2022)
	Null	Null	Null		Null	Null	ICESat	8-10	Kääb et al. (2015)
Hengduan	Null	Null	Null		2.39 ± 0.18<br>1.13 ± 0.84	Cao et al. (2021)<br>Li et al. (2021)<br>Fan et al. (2023)	Null	Null	Null
Eastern Himalaya	Null	Null	Null		1.0<br>3.15 ± 0.17<br>1.13 ± 0.56	Zhou et al (2018)<br>Li et al. (2021)<br>Fan et al. (2023)	ICESat	4.8 ± 0.7 (s)<br>0.1 ± 1.2 (i)<br>2.5 ± 0.5	Kääb et al. (2015)
Central Himalaya	TanDEM-X Pléiades	0.68 ± 0.28(i)<br>0.24 ± 0.18(d)	Bhattacharya et al. (2021)		1.4 (CN)<br>1.9 (WN)<br>3.15 ± 0.17<br>1.85 ± 0.69	Zhou et al (2018)<br>Li et al. (2021)<br>Fan et al. (2023)	ICESat	2.3 ± 0.6 (s)<br>1.7 ± 0.6 (i)<br>1.5 ± 0.4	Kääb et al. (2015)
Western Himalaya	Null	Null	Null		1.3<br>3.04 ± 0.23<br>1.59 ± 0.57	Zhou et al (2018)<br>Li et al. (2021)<br>Fan et al. (2023)	ICESat	2.3 ± 0.6 (s)<br>1.7 ± 0.6 (i)<br>1.5 ± 0.4	Kääb et al. (2015)

Table S6. X/C-band penetration depth and their differences we used in this study. Note that the bold in the second and third column denotes the estimated results.

Region	X-band penetration depth (m)	Penetration depth difference (m)	C-band penetration depth (m)
Eastern Tianshan	1.54	**1.41**	2.95
Central Tianshan	1.54	**1.41**	2.95
Western Tianshan	1.54	**1.41**	2.95
Dzhungarsky Alatau	1.54	**1.41**	2.95
Pamir Alay	1.54	**1.41**	2.95
Eastern Pamir	2	**1.15**	3.15
Western Pamir	2	**1.15**	3.15
Karakoram	**2**	**1.57**	3.57
Hindu Kush	2	**1.04**	3.04
Western Kunlun	**2.10**	**2.05**	3.15
Eastern Kunlun	1.54	**1.75**	3.29
Altun	1.54	**1.75**	3.29
Qilian	1.54	**1.91**	3.45
Qiangtang Plateau	**1.56**	**2.21**	3.77
Tanggula	**1.52**	**2.21**	3.73
Eastern Tibet	1.54	**1.27**	2.81
Gangdise	1.56	**2.21**	3.77
Nyainqentanglha	**1.84**	**1.76**	3.60
Hengduan	1.54	**1.76**	3.30
Eastern Himalaya	1.92	**1.07**	2.99
Central Himalaya	1.92	**1.72**	3.64
Western Himalaya	1.92	**1.45**	3.37

Table S7. Model Subset Performance Ranked by AIC for westerly region (Lower AIC Values Indicate Superior Fit)

Predictors	AIC	BIC	Adjusted R2	RMSE
**Liquid_precipitation + Solid_precipitation + Summer_temperature**	**121.38**	**122.97**	**0.7782**	**52.49**
Liquid_precipitation + Solid_precipitation + Annual_temperature	121.6	123.19	0.7737	53.019
Liquid_precipitation + Annual_temperature	121.66	122.85	0.76121	54.463
Liquid_precipitation + Solid_precipitation	122.17	123.36	0.74994	55.733
Liquid_precipitation + Solid_precipitation + Annual_temperature +Summer_temperature	122.94	124.93	0.75135	55.576
Liquid_precipitation + Summer_temperature	123.03	124.22	0.72958	57.958
Liquid_precipitation	123.06	123.86	0.71071	59.946
Liquid_precipitation + Annual_temperature + Summer_temperature	123.55	125.14	0.72986	57.928
Solid_precipitation + Summer_temperature	131.32	132.52	0.42511	84.505
Solid_precipitation	131.67	132.46	0.36767	88.626
Solid_precipitation + Annual_temperature	132.59	133.78	0.35499	89.511
Solid_precipitation + Annual_temperature + Summer_temperature	133.32	134.92	0.34298	90.34
Annual_temperature	135.52	136.32	0.10222	105.6
Summer_temperature	135.92	136.71	0.069469	107.51
Annual_temperature + Summer_temperature	137.25	138.45	0.014381	110.65

Table S8. Model Subset Performance Ranked by AIC for monsoon region (Lower AIC Values Indicate Superior Fit)

Predictors	AIC	BIC	Adjusted R2	RMSE
**Liquid_precipitation + Solid_precipitation + Annual_temperature + Summer_temperature**	**87.90**	**88.30**	**0.89**	**51.44**
Solid_precipitation + Annual_temperature + Summer_temperature	88.88	89.20	0.88	53.67
Solid_precipitation + Annual_temperature	92.03	92.27	0.81	66.23
Liquid_precipitation + Solid_precipitation + Annual_temperature	92.97	93.29	0.80	69.31
Solid_precipitation	96.56	96.72	0.65	90.91
Solid_precipitation + Summer_temperature	97.72	97.96	0.62	94.50
Liquid_precipitation + Solid_precipitation	98.48	98.72	0.58	99.07
Liquid_precipitation + Annual_temperature + Summer_temperature	99.09	99.41	0.56	101.56
Annual_temperature + Summer_temperature	99.29	99.53	0.54	104.24
Liquid_precipitation + Solid_precipitation + Summer_temperature	99.72	100.04	0.53	105.65
Liquid_precipitation + Annual_temperature	100.34	100.58	0.47	111.34
Liquid_precipitation	100.72	100.88	0.41	117.92
Annual_temperature	101.46	101.62	0.35	123.51
Liquid_precipitation + Summer_temperature	102.67	102.91	0.30	128.76
Summer_temperature	105.87	106.03	-0.12	162.72

Supplementary References

1. Azam, M. F., Ramanathan, A., Wagnon, P., Vincent, C., Linda, A., Berthier, E., Sharma, P., Mandal, A., Angchuk, T., & Pottakkal, J. G. (2016). Meteorological conditions, seasonal and annual mass balances of Chhota Shigri Glacier, western Himalaya, India. Annals of Glaciology, 57(71), 328-338.
2. Azam, M. F., Wagnon, P., Ramanathan, A., Vincent, C., Sharma, P., Arnaud, Y., Linda, A., Pottakkal, J. G., Berthier, E., & Shea, J. M. (2012). From balance to imbalance: a shift in the dynamic behaviour of Chhota Shigri glacier, western Himalaya, India. Journal of Glaciology, 58(208), 315-324.
3. Azam, M. F., Wagnon, P., Vincent, C., Ramanathan, A., Favier, V., Mandal, A., & Pottakkal, J. G. (2014a). Reconstruction of the annual mass balance of Chhota Shigri glacier, Western Himalaya, India, since 1969. Annals of Glaciology, 55(66), 69-80.
4. Azam, M. F., Wagnon, P., Vincent, C., Ramanathan, A., Linda, A., & Singh, V. B. (2014b). Processes governing the mass balance of Chhota Shigri Glacier (western Himalaya, India) assessed by point-scale surface energy balance measurements. The Cryosphere, 8(6), 2195-2217.
5. Barandun, M., Huss, M., Sold, L., Farinotti, D., Azisov, E., Salzmann, N., ... & Hoelzle, M. (2015). Re-analysis of seasonal mass balance at Abramov glacier 1968–2014. *Journal of Glaciology*, *61*(230), 1103-1117.
6. Barandun, M., Pohl, E., Naegeli, K., McNabb, R., Huss, M., Berthier, E., ... & Hoelzle, M. (2021). Hot spots of glacier mass balance variability in Central Asia. *Geophysical Research Letters*, *48*(11), e2020GL092084.
7. Baral, P., Kayastha, R. B., Immerzeel, W. W., Pradhananga, N. S., Bhattarai, B. C., Shahi, S., Galos, S., Springer, C., Joshi, S. P., & Mool, P. K. (2014). Preliminary results of mass-balance observations of Yala Glacier and analysis of temperature and precipitation gradients in Langtang Valley, Nepal. Annals of Glaciology, 55(66), 9-14.
8. Bhattacharya, A., Bolch, T., Mukherjee, K., King, O., Menounos, B., Kapitsa, V., ... & Yao, T. (2021). High Mountain Asian glacier response to climate revealed by multi-temporal satellite observations since the 1960s. *Nature communications*, *12*(1), 4133.
9. Bolch, T., Pieczonka, T., Mukherjee, K., & Shea, J. (2017). Brief communication: Glaciers in the Hunza catchment (Karakoram) have been nearly in balance since the 1970s. *The Cryosphere*, *11*(1), 531-539.
10. Bolch, T.. Shea, J.M., Liu, S., Azam, F.M., Gao, Y., Gruber, S. et al. (2019). Status and change of the cryosphere in the extended Hindu Kush Himalaya region, in *The Hindu Kush Himalaya Assessment: Mountains, Climate Change, Sustainability and People*, eds Wester P., Mishra A., Mukherji A., Shrestha A.B. (Cham: Springer), 209–255. 10.1007/978-3-319-92288-1_7
11. Brun, F., Berthier, E., Wagnon, P., Kääb, A., & Treichler, D. (2017). A spatially resolved estimate of High Mountain Asia glacier mass balances from 2000 to 2016. *Nature*

geoscience, *10*(9), 668-673.

12. Cao, B. O., Pan, B., Guan, W., Wen, Z., & Wang, J. I. E. (2019). Changes in glacier volume on Mt. Gongga, southeastern Tibetan Plateau, based on the analysis of multi-temporal DEMs from 1966 to 2015. *Journal of Glaciology*, *65*(251), 366-375.
13. Chen, W., Yao, T., Zhang, G., Li, S., & Zheng, G. (2021). Accelerated glacier mass loss in the largest river and lake source regions of the Tibetan Plateau and its links with local water balance over 1976–2017. *Journal of Glaciology*, *67*(264), 577-591.
14. Dussaillant, I., Hugonnet, R., Huss, M., Berthier, E., Bannwart, J., Paul, F., & Zemp, M. (2025). Annual mass change of the world's glaciers from 1976 to 2024 by temporal downscaling of satellite data with in situ observations. *Earth System Science Data*, *17*(5), 1977-2006.
15. Denzinger, F., Machguth, H., Barandun, M., Berthier, E., Girod, L., Kronenberg, M., ... & Hoelzle, M. (2021). Geodetic mass balance of Abramov Glacier from 1975 to 2015. *Journal of Glaciology*, *67*(262), 331-342.
16. Dobhal, D. P., Gergan, J. T., & Thayyen, R. J. (2008). Mass balance studies of the Dokriani Glacier from 1992 to 2000, Garhwal Himalaya, India. Bulletin of Glaciological Research, 25, 9-17.
17. Dobhal, D. P., Mehta, M., & Srivastava, D. (2013). Influence of debris cover on terminus retreat and mass changes of Chorabari Glacier, Garhwal region, central Himalaya, India. Journal of Glaciology, 59(217), 961-971.
18. Fan, Y., Ke, C. Q., Zhou, X., Shen, X., Yu, X., & Lhakpa, D. (2023). Glacier mass-balance estimates over High Mountain Asia from 2000 to 2021 based on ICESat-2 and NASADEM. *Journal of Glaciology*, *69*(275), 500-512.
19. Fujita, K., Kadota, T., Rana, B., Kayastha, R. B., & Ageta, Y. (2001a). Shrinkage of Glacier AX010 in Shorong region, Nepal Himalayas in the 1990s. Bulletin of Glaciological Research, 18, 51-54.
20. Fujita, K., Nakazawa, F., & Rana, B. (2001b). Glaciological observations on Rikha Samba Glacier in Hidden Valley, Nepal Himalayas, 1998 and 1999. Bulletin of Glaciological Research, 18, 31-35.
21. Gardelle, J., Berthier, E., Arnaud, Y., & Kääb, A. (2013). Region-wide glacier mass balances over the Pamir-Karakoram-Himalaya during 1999–2011. *The Cryosphere*, *7*(4), 1263-1286.
22. Gautam, C. K., & Mukherjee, B. P. (1992). Synthesis of glaciological studies on Tipra Bank Glacier, Bhyundar Ganga Basin, district Chamoli, Uttar Pradesh (F.S. 1980-1988). Geological Survey of India, Northern Region, Lucknow.
23. Goerlich, F., Bolch, T., Mukherjee, K., & Pieczonka, T. (2017). Glacier mass loss during the 1960s and 1970s in the Ak-Shirak range (Kyrgyzstan) from multiple stereoscopic Corona and Hexagon imagery. *Remote Sensing*, *9*(3), 275.
24. GSI (Geological Survey of India). (1977). Report of the interdepartmental expedition to Gara Glacier 1975. Geological Survey of India, Northern Region, Calcutta.

25. GSI (Geological Survey of India). (1991). Annual general report, Part 8, Volume 124. Geological Survey of India.
26. GSI (Geological Survey of India). (1992). Annual general report, Part 8, Volume 125. Geological Survey of India.
27. GSI (Geological Survey of India). (2001). Glaciology of Indian Himalaya (Special Publication No. 63). Geological Survey of India.
28. GSI (Geological Survey of India). (2011). Annual general report, Part 8, Volume 144. Geological Survey of India.
29. GTN-G (2023): GTN-G Glacier Regions. Global Terrestrial Network for Glaciers. DOI: 10.5904/gtng-glacreg-2023-07. Online access: https://dx.doi.org/10.5904/gtng-glacreg-2023-07.
30. Hugonnet, R., McNabb, R., Berthier, E., Menounos, B., Nuth, C., Girod, L., ... & Kääb, A. (2021). Accelerated global glacier mass loss in the early twenty-first century. Nature, 592(7856), 726-731.
31. Hugonnet, R. et al. Uncertainty Analysis of Digital Elevation Models by Spatial Inference From Stable Terrain. IEEE Journal of Selected Topics in Applied Earth Observations and Remote Sensing 15, 6456-6472 (2022)
32. Holzer, N., Vijay, S., Yao, T., Xu, B., Buchroithner, M., & Bolch, T. (2015). Four decades of glacier variations at Muztagh Ata (eastern Pamir): a multi-sensor study including Hexagon KH-9 and Pléiades data. The Cryosphere, 9(6), 2071-2088.
33. Huss, M. Density assumptions for converting geodetic glacier volume change to mass change. The Cryosphere 7, 877-887 (2013)
34. Kääb, A., Berthier, E., Nuth, C., Gardelle, J., & Arnaud, Y. (2012). Contrasting patterns of early twenty-first-century glacier mass change in the Himalayas. *Nature*, *488*(7412), 495-498.
35. Kääb, A., Treichler, D., Nuth, C., & Berthier, E. J. T. C. (2015). Brief Communication: Contending estimates of 2003–2008 glacier mass balance over the Pamir–Karakoram–Himalaya. *The Cryosphere*, *9*(2), 557-564.
36. Ke, L., Song, C., Wang, J., Sheng, Y., Ding, X., Yong, B., ... & Luo, S. (2022). Constraining the contribution of glacier mass balance to the Tibetan lake growth in the early 21st century. *Remote Sensing of Environment*, *268*, 112779.
37. King, O., Bhattacharya, A., Bhambri, R., & Bolch, T. (2019). Glacial lakes exacerbate Himalayan glacier mass loss. *Scientific Reports*, *9*(1), 18145.
38. Kronenberg, M., van Pelt, W., Machguth, H., Fiddes, J., Hoelzle, M., & Pertziger, F. (2022). Long-term firn and mass balance modelling for Abramov Glacier in the data-scarce Pamir Alay. *The Cryosphere*, *16*(12), 5001-5022.
39. Kumar, A., Negi, H. S., Kumar, K., Shekhar, C., & Kanda, N. (2019). Quantifying mass balance of East-Karakoram glaciers using geodetic technique. *Polar Science*, *19*, 24-39.
40. Kaul, M. N. (1986). Mass balance of Liddar glaciers. Transactions of the Institute of Indian

Geographers, 8, 95-111.

41. Koul, M. N., & Ganjoo, R. K. (2010). Impact of inter- and intra-annual variation in weather parameters on mass balance and equilibrium line altitude of Naradu Glacier (Himachal Pradesh), NW Himalaya, India. Climatic Change, 99(1), 119-139.
42. Kumar, R., Singh, S., Kumar, R., Singh, A., Bhardwaj, A., Sam, L., & Gupta, A. (2014). Mass and energy balance study of Naradu Glacier, Himachal Himalaya. National Conference of Himalayan Glaciology, Shimla, India.
43. Lambrecht, A., Mayer, C., Wendt, A., Floricioiu, D., & Völksen, C. (2018). Elevation change of Fedchenko Glacier, Pamir Mountains, from GNSS field measurements and TanDEM-X elevation models, with a focus on the upper glacier. *Journal of Glaciology*, *64*(246), 637-648.
44. Li, C., Jiang, L., Liu, L., & Wang, H. (2021). Regional and altitude-dependent estimate of the SRTM C/X-band radar penetration difference on High Mountain Asia glaciers. *IEEE Journal of Selected Topics in Applied Earth Observations and Remote Sensing*, *14*, 4244-4253.
45. Li, C., Jiang, L., Liu, L., Li, T., Chen, Y. (2023). Multi-period mass balance estimation of glaciers in the western Qilian Mountains based on the combination of bistatic SAR DEM and WordView DEM. *National Remote Sensing Bulletin*, *27*(9), 2085-2097.
46. Li, G., & Lin, H. (2017). Recent decadal glacier mass balances over the Western Nyainqentanglha Mountains and the increase in their melting contribution to Nam Co Lake measured by differential bistatic SAR interferometry. *Global and Planetary Change*, *149*, 177-190.
47. Li, J., Li, Z. W., Zhu, J. J., Li, X., Xu, B., Wang, Q. J., ... & Hu, J. (2017). Early 21st century glacier thickness changes in the Central Tien Shan. *Remote Sensing of Environment*, *192*, 12-29.
48. Li, J., Li, Z. W., Hu, J., Wu, L. X., Li, X., Guo, L., ... & Chen, J. L. (2021). Investigating the bias of TanDEM-X digital elevation models of glaciers on the Tibetan Plateau: impacting factors and potential effects on geodetic mass-balance measurements. *Journal of Glaciology*, *67*(264), 613-626.
49. Li, J. et al. Investigating the bias of TanDEM-X digital elevation models of glaciers on the Tibetan Plateau: impacting factors and potential effects on geodetic mass-balance measurements. Journal of Glaciology 67, 613-626 (2021)
50. Lin, H., Li, G., Cuo, L., Hooper, A., & Ye, Q. (2017). A decreasing glacier mass balance gradient from the edge of the Upper Tarim Basin to the Karakoram during 2000–2014. *Scientific reports*, *7*(1), 6712.
51. Liu, L., Jiang, L., Jiang, H., Wang, H., Ma, N., & Xu, H. (2019). Accelerated glacier mass loss (2011–2016) over the Puruogangri ice field in the inner Tibetan Plateau revealed by bistatic InSAR measurements. *Remote Sensing of Environment*, *231*, 111241.
52. Liu, S., Xie, Z., Song, G., Ma, L., & Ageta, Y. (1996). Mass balance of Kangwure (flat-top) Glacier on the north side of Mt. Xixiabangma, China. Bulletin of Glacier Research, 14, 37-43.

53. Lv, M., Quincey, D. J., Guo, H., King, O., Liu, G., Yan, S., ... & Ruan, Z. (2020). Examining geodetic glacier mass balance in the eastern Pamir transition zone. *Journal of Glaciology*, *66*(260), 927-937.
54. Maurer, J. & Rupper, S. Tapping into the Hexagon spy imagery database: A new automated pipeline for geomorphic change detection. ISPRS Journal of Photogrammetry and Remote Sensing 108, 113-127 (2015).
55. Maurer, J. M., Schaefer, J. M., Rupper, S., & Corley, A. J. S. A. (2019). Acceleration of ice loss across the Himalayas over the past 40 years. *Science advances*, *5*(6), eaav7266.
56. Mannerfelt, E. S., Dehecq, A., Hugonnet, R., Hodel, E., Huss, M., Bauder, A., & Farinotti, D. (2022). Halving of Swiss glacier volume since 1931 observed from terrestrial image photogrammetry. *The Cryosphere*, *16*(8), 3249-3268.
57. Mou, J. X., Li, Z. Q., Zhang, H., Xu, C. H., Jin, S., & Liang, P. B. (2019). Changes in mass balance of continental and maritime glaciers in western China and their climatic responses: A case study of Urumqi Glacier No. 1 and Parlung Glacier No. 94. Arid Land Geography, 42 (1), 20-28.
58. Mishra, R., Kumar, A., & Singh, D. (2014). Long term monitoring of mass balance of Hamtah Glacier, Lahaul
59. Nuth, C. & Kääb, A. Co-registration and bias corrections of satellite elevation data sets for quantifying glacier thickness change. The Cryosphere 5, 271-290 (2011).
60. Pieczonka, T., Bolch, T., Junfeng, W., & Shiyin, L. (2013). Heterogeneous mass loss of glaciers in the Aksu-Tarim Catchment (Central Tien Shan) revealed by 1976 KH-9 Hexagon and 2009 SPOT-5 stereo imagery. *Remote Sensing of Environment*, *130*, 233-244.
61. Pieczonka, T., & Bolch, T. (2015). Region-wide glacier mass budgets and area changes for the Central Tien Shan between~ 1975 and 1999 using Hexagon KH-9 imagery. *Global and Planetary Change*, *128*, 1-13.
62. Raina, V. K., Kaul, M. K., & Singh, S. (1977). Mass-balance studies of Gara Glacier. Journal of Glaciology, 18(80), 415-423.
63. Ren, P., Pan, X., Liu, T., Huang, Y., Chen, X., Wang, X., ... & Akmalov, S. (2024). Glacier changes from 1990 to 2022 in the Aksu River Basin, western Tien Shan. *Remote Sensing*, *16*(10), 1751.
64. Ren, S., Menenti, M., Jia, L., Zhang, J., Zhang, J., & Li, X. (2020). Glacier mass balance in the Nyainqentanglha Mountains between 2000 and 2017 retrieved from ZiYuan-3 stereo images and the SRTM DEM. *Remote Sensing*, *12*(5), 864.
65. Ren, S., Li, X., Wang, Y., Zheng, D., Jiang, D., Nian, Y., & Zhou, Y. (2022). Multitemporal glacier mass balance and area changes in the Puruogangri Ice Field during 1975–2021 based on multisource satellite observations. *Remote Sensing*, *14*(16), 4078.
66. RGI 7.0 Consortium, 2023. Randolph Glacier Inventory - A Dataset of Global Glacier Outlines, Version 7.0. Boulder, Colorado USA. NSIDC: National Snow and Ice Data Center.

doi:10.5067/f6jmovy5navz. Online access: https://doi.org/10.5067/f6jmovy5navz https://doi.org/10.5067/f6jmovy5navz

67. Round, V., Leinss, S., Huss, M., Haemmig, C., & Hajnsek, I. (2017). Surge dynamics and lake outbursts of Kyagar Glacier, Karakoram. *The Cryosphere*, *11*(2), 723-739.
68. Sangewar, C. V., & Sangewar, M. A. (2007). Thematic compilation of mass balance data on glaciers of Satluj Catchment in Himachal Himalaya (Field Season: 2006-07). Geological Survey of India, Northern Region, Lucknow.
69. Sherpa, S. F., Wagnon, P., Brun, F., Berthier, E., Vincent, C., Lejeune, Y., Arnaud, Y., Kayastha, R. B., & Sinisalo, A. (2017). Contrasted surface mass balances of debris-free glaciers observed between the southern and the inner parts of the Everest region (2007-15). Journal of Glaciology, 63(240), 637-651.
70. Shrivastava, D., Sangewar, C. V., Kaul, M. K., & Jamwal, K. S. (2001). Mass balance of Rulung Glacier—A Trans-Himalayan glacier, Indus basin, Ladakh. Proceedings of the Symposium on Snow, Ice and Glacier, March 1999. Geological Survey of India, Special Publication, 53, 41-46.
71. Su, B., Li, Z.Q., Zhang, M. J., Guo, R., Sun, M. P., Che, Y. J., Ying, X. (2015). Comparative study on mass balance of continental and maritime glaciers: A case study of typical glaciers in the Tien Shan and the Alps. Journal of Glaciology and Geocryology, 37(5), 1131-1140.
72. Shean, D. E., Bhushan, S., Montesano, P., Rounce, D. R., Arendt, A., & Osmanoglu, B. (2020). A systematic, regional assessment of high mountain Asia glacier mass balance. *Frontiers in Earth Science*, *7*, 363.
73. Sun, Y., Jiang, L., Liu, L., Sun, Q., Wang, H., & Hsu, H. (2017). Mapping glacier elevations and their changes in the western Qilian Mountains, northern Tibetan Plateau, by bistatic InSAR. IEEE Journal of Selected Topics in Applied Earth Observations and Remote Sensing, 11(1), 68-78.
74. Tshering, P., & Fujita, K. (2016). First in situ record of decadal glacier mass balance (2003-2014) from the Bhutan Himalaya. Annals of Glaciology, 57(71), 289-294.
75. Vincent, C., Ramanathan, A., Wagnon, P., Dobhal, D. P., Linda, A., Berthier, E., Sharma, P., Arnaud, Y., Azam, M. F., & Gardelle, J. (2013). Balanced conditions or slight mass gain of glaciers in the Lahaul and Spiti region (northern India, Himalaya) during the nineties preceded recent mass loss. The Cryosphere, 7(2), 569-582.
76. Wang, W., Li, Z., Zhang, G., Li, X. (2012). The processes and characteristics of mass balance on the Urumqi Glacier No. 1 during 1958-2009. Sciences in Cold and Arid Regions, 4(06), 505-513
77. Wagnon, P., Linda, A., Arnaud, Y., Kumar, R., Sharma, P., Vincent, C., Pottakkal, J. G., Berthier, E., Ramanathan, A., & Chevallier, P. (2007). Four years of mass balance on Chhota Shigri Glacier, Himachal Pradesh, India, a new benchmark glacier in the western Himalaya. Journal of Glaciology, 53(183), 603-611.

78. Wagnon, P., Vincent, C., Arnaud, Y., Berthier, E., Vuillermoz, E., Gruber, S., Ménégoz, M., Gilbert, A., Dumont, M., Shea, J. M., & Pokhrel, B. K. (2013). Seasonal and annual mass balances of Mera and Pokalde glaciers (Nepal Himalaya) since 2007. The Cryosphere, 7(6), 1769-1786.
79. Wei, L. (2025). Characteristics of mass balance changes in mountain glaciers and study on key influencing factors [Master's thesis, Lanzhou University].
80. Wu, K., Liu, S., Jiang, Z., Xu, J., Wei, J., & Guo, W. (2018). Recent glacier mass balance and area changes in the Kangri Karpo Mountains from DEMs and glacier inventories. The Cryosphere, 12(1), 103-121.
81. Wu, K., Liu, S., Jiang, Z., Xu, J., & Wei, J. (2019). Glacier mass balance over the central Nyainqentanglha Range during recent decades derived from remote-sensing data. *Journal of Glaciology*, *65*(251), 422-439.
82. Yao, T., Thompson, L., Yang, W., Yu, W., Gao, Y., Guo, X., Yang, X., Duan, K., Zhao, H., Xu, B., & Joswiak, D. (2012). Different glacier status with atmospheric circulations in Tibetan Plateau and surroundings. Nature Climate Change, 2, 663-667.
83. Zhang, G., Chen, W., Li, G., Yang, W., Yi, S., & Luo, W. (2020). Lake water and glacier mass gains in the northwestern Tibetan Plateau observed from multi-sensor remote sensing data: Implication of an enhanced hydrological cycle. Remote Sensing of Environment, 237, 111554.
84. Zhang, H., Li, Z., Zhou, P., Zhu, X., & Wang, L. I. N. (2018). Mass-balance observations and reconstruction for Haxilegen Glacier No. 51, eastern Tien Shan, from 1999 to 2015. *Journal of Glaciology*, *64*(247), 689-699.
85. Zhang, H. (2022). Study on glacier mass balance changes on the northern slope of the Qilian Mountains and their contribution to runoff [Master's thesis, Northwest Normal University].
86. Zhang, Z., Liu, S., Jiang, Z., Shangguan, D., Wei, J., Guo, W., ... & Huang, D. (2020). Glacier variations at Xinqingfeng and Malan ice caps i the Inner Tibetan Plateau since 1970. *Remote Sensing*, *12*(3), 421.
87. Zhou, Y., Li, Z., & Li, J. I. A. (2017). Slight glacier mass loss in the Karakoram region during the 1970s to 2000 revealed by KH-9 images and SRTM DEM. *Journal of Glaciology*, *63*(238), 331-342.
88. Zhou, Y., Li, Z., Li, J., Zhao, R. & Ding, X. Glacier mass balance in the Qinghai–Tibet Plateau and its surroundings from the mid-1970s to 2000 based on Hexagon KH-9 and SRTM DEMs. Remote Sensing of Environment 210, 96-112 (2018).
89. Zhou, Y., Li, Z., Li, J. I. A., Zhao, R., & Ding, X. (2019). Geodetic glacier mass balance (1975–1999) in the central Pamir using the SRTM DEM and KH-9 imagery. *Journal of Glaciology*, *65*(250), 309-320.
90. Zhou, Y., Li, X., Zheng, D., & Li, Z. (2022). Evolution of geodetic mass balance over the largest lake-terminating glacier in the Tibetan Plateau with a revised radar penetration depth based on multi-source high-resolution satellite data. *Remote Sensing of Environment*, *275*,

113029.
91. Zhou, Y., Li, X., Zheng, D., Zhang, X., Wang, Y., Ren, S., & Guo, Y. (2022). Decadal changes in glacier area, surface elevation and mass balance for 2000–2020 in the eastern tanggula mountains using optical images and tandem-x radar data. Remote Sensing, 14(3), 506.
